# Systems approaches to scaling up: a systematic review and narrative synthesis of evidence for physical activity and other behavioural non-communicable disease risk factors

**DOI:** 10.1186/s12966-024-01579-6

**Published:** 2024-03-21

**Authors:** Harriet Koorts, Jiani Ma, Christopher T. V. Swain, Harry Rutter, Jo Salmon, Kristy A. Bolton

**Affiliations:** 1 School of Exercise and Nutrition Sciences, Deakin University, Institute for Physical Activity and Nutrition (IPAN), 221 Burwood Highway, Burwood Geelong, VIC 3125 Australia; 2 Cancer Epidemiology Division, Cancer Council Victoria, Melbourne, Australia; 3 Department of Physiotherapy, Faculty of Medicine Dentistry and Health Sciences, Melbourne School of Health Sciences, University of Melbourne, Melbourne, Australia; 4 Department of Social and Policy Sciences, University of Bath, Claverton Down, Bath, UK

**Keywords:** System, Public health, Scale up, Implementation, Nutrition, Tobacco, Alcohol, Diet, Non-communicable diseases

## Abstract

**Background:**

Non-communicable diseases (NCDs) are the leading causes of death worldwide. Systems approaches have potential for creating sustainable outcomes at scale but have rarely been used to support scale up in physical activity/nutrition promotion or NCD prevention more generally. This review aimed to: (i) synthesise evidence on the use of systems approaches in scaling up interventions targeting four behavioural risk factors for NCDs; and (ii) to explore how systems approaches have been conceptualised and used in intervention implementation and scale up.

**Method:**

Seven electronic databases were searched for studies published 2016–2021. Eligible studies targeted at least one of four NCD behavioural risk factors (physical inactivity, tobacco use, alcohol consumption, diet), or described evaluation of an intervention planned for or scaled up. Studies were categorised as having a (i) *high*, (ii) *moderate*, or (iii) *no* use of a systems approach. A narrative synthesis of how systems approaches had been operationalised in scale up, following PRISMA guidelines.

**Results:**

Twenty-one intervention studies were included. Only 19% (*n* = 4) of interventions explicitly used systems thinking to inform intervention design, implementation and scale up (targeting all four risk factors *n* = 2, diet *n* = 1, tobacco use *n* = 1). Five studies (‘high use’) planned and implemented scale up with an explicit focus on relations between system elements and used system changes to drive impact at scale. Seven studies (‘moderate use’) considered systems elements impacting scale-up processes or outcomes but did not require achieving system-level changes from the outset. Nine studies (‘no use’) were designed to work at multiple levels among multiple agencies in an intervention setting, but the complexity of the system and relations between system elements was not articulated. We synthesised reported barriers and facilitators to scaling up, and how studies within each group conceptualised and used systems approaches, and methods, frameworks and principles for scaling up.

**Conclusion:**

In physical activity research, and NCD prevention more broadly, the use of systems approaches in scale up remains in its infancy. For researchers, practitioners and policymakers wishing to adopt systems approaches to intervention implementation at scale, guidance is needed on how to communicate and operationalise systems approaches in research and in practice.

**Trial registration:**

PROSPERO (CRD42021287265).

**Supplementary Information:**

The online version contains supplementary material available at 10.1186/s12966-024-01579-6.

## Introduction

Non-communicable diseases (NCDs), such as cardiovascular disease, cancer and type 2 diabetes, are the leading causes of death worldwide, contributing to more than 41 million deaths globally [1]. Major behavioural risk factors for NCDs include physical inactivity, tobacco use, alcohol consumption and an unhealthy diet [2]. The high burden of NCDs causes substantial economic losses worldwide, and deaths from NCDs disproportionately affect low- and middle-income countries [1]. Whilst there are multiple targeted action plans for the prevention of NCDs, such as the Global Action Plan for the Prevention and Control of Non-communicable Diseases 2013–2030 [2], and for specific risk factors such as the Global Action Plan for Physical Activity (GAPPA) [3] and Global Alcohol Action Plan 2022–2030 [4]; many of the NCD risk factors have reached pandemic proportions. For example, annually, tobacco use causes over 8 million deaths https://www.who.int/news-room/fact-sheets/detail/tobacco, around 3.2 million deaths are attributed to physical inactivity [5], over 3 million deaths result from the harmful use of alcohol [6], and 2.8 million deaths are as a result of being overweight or obese [7]. By 2030, global health-care costs of physical inactivity alone are estimated to exceed INT$520 billion [8]. Increasing implementation of evidence-based solutions to reduce the risk of NCDs at scale is a thus global priority of the World Health Organization (WHO) [9]. Despite burgeoning evidence for the effectiveness of different interventions to prevent NCDs; researchers, practitioners and policymakers have limited access to effective ways of *scaling up* NCD risk factor interventions, which are essential for global shifts in health [10, 11].

Scaling up presents a complex set of challenges. They are complex, not only due to the factors underpinning NCD risk factors, but also in the nature of the processes required to achieve impact at large scale. Interventions intended for scale up should thus be planned with consideration of complexity [12–14]. Addressing one element within a complex public health problem (e.g., through a discrete intervention targeting a specific factor) is unlikely to achieve desirable population-wide effects at scale [10]. For physical inactivity, intervention effects can be attenuated at scale [15], and yet the mechanisms underpinning outcomes of scaling up, which may contribute to these attenuated effects, are often complex and poorly understood [16]. Large-scale interventions span many different community contexts and adapted in response to these contexts, posing problems for the attribution of impact during evaluation [12]. The scaling process itself often involves multiple delivery strategies or systems, and the vast number of settings, contexts and systems affected during scale-up can extend beyond the capacity for data collection [13]. This lack of knowledge of the complex interactions between factors when scaling up poses difficulties when generalising ‘effective’ approaches to successful scaling.

Systems approaches provide a framework for exploring the multitude of interdependent elements that influence a problem, and the scaling up of solutions for that problem, as they can help establish the relations between factors, how they change over time, and acknowledge that effective actions are required across political, social, cultural, economic and scientific domains within the system [17]. All the NCD risk factors themselves have a multitude of interdependent elements that are complex, interconnected and have interacting influences [18–20]. Traditional, linear ‘blueprint approaches’ to scaling up that are observed in global health initiatives may not adequately fit the dynamic and unpredictable ways in which health services, organisations, and communities expand and are sustained [21].

Complexity and systems theory can make a valuable contribution to understanding and addressing population health scale up [13]. Systems approaches are also theorised to be influential in creating sustainable outcomes at scale [13, 14]. However, in public health generally, there is limited evidence for the impacts of adopting a systems approach, with a paucity of detailed descriptions of their operationalisation. In health systems research, a meta-analysis of 35 studies identified a significant improvement to patient and service outcomes when a systems approach informed healthcare design and delivery [22]. However, in NCD behavioural risk factor research, systems approaches have had mixed adoption. For example, in tobacco cessation research, for almost 30 years systems approaches have been recognised and systems level strategies applied in health services [23], whereas for physical activity there are few well described examples of interventions that have been planned, delivered and evaluated using systems approaches and systems analysis methods [24].

For researchers, practitioners and policymakers wishing to achieve population level impact of interventions, and adopt a systems approach to intervention implementation at scale, greater guidance is needed on ways to achieve this and how to operationalise systems approaches in the context of public health scale up. Given that in public health generally, evidence to demonstrate the value of applying a systems approach is still emerging [25], and there is little research that has examined systems-based practice [26] or how systems approaches are applied in scaling up in public health [13, 14]; we sought to contribute to addressing these gaps in the current review.

The objective of this paper is to synthesise the evidence of how systems approaches have been used to inform scaling up in physical activity. However, to explore scaling up of interventions targeting behavioural risk factors for NCDs more comprehensively, the scope was broadened to include the three other key NCD behavioural risk factors: tobacco use, alcohol consumption, and diet. As NCD prevention often operationalises ‘diet’ in terms of obesity, we consider diet and obesity jointly; herein ‘diet/obesity’. Given the current lack of published evidence describing implementation of systems approaches in public health [27], and the fact that interventions targeting physical activity are often designed as multicomponent interventions targeting multiple NCD risk factors combined (i.e., physical activity and diet), this strategy also provides an opportunity to learn from other areas of public health.

The specific aims of this review are as follows. Firstly, to identify how systems approaches have been used to inform and understand: (i) approaches and strategies to scaling up interventions targeting four behavioural risk factors for NCD; and (ii) barriers and facilitators to scaling up of these interventions. Secondly, how the term ‘systems’ has been conceptualised and used in the broader context of intervention implementation and scale up in these studies will be identified.

## Methods

This review was prospectively registered with PROSPERO (registration number CRD42021287265) and follows the Preferred Reporting Items for Systemic Reviews and Meta-Analyses (PRISMA) guidelines [28]. The PRISMA checklist is presented in Additional file 1.

### Definition of a systems approach to scaling up

Systems-based public health is an evolving field, with no widely agreed definition of what it entails [22, 29]. A ‘systems approach’, however, is generally understood as an approach that takes into account a multiplicity of interacting factors across a system, and the ways in which that system responds and adapts to interventions within it [17]. ‘Scale up’ refers to “*deliberate efforts to increase the impact of successfully tested interventions, to benefit a greater number of people and to foster policy and programme development”* [30]. Whilst scale up processes commence once an intervention has been developed, in practice settings, scale up can occur before an intervention has been tested within a research trial [31]. ‘Dissemination’ is another term often used when referring to at-scale implementation, as it refers to an active approach to spreading an evidence-based intervention using planned strategies [32]. However, unlike ‘scale up’, dissemination need not include efforts to maximise the *scale* of interventions. More recently, a ‘systems approach to scale up’ has been defined as an approach that “*prioritises the behaviour and function of the system, with a focus on relations between a number of system elements, using system-level levers and dynamic system changes to drive impact at scale*” [14]. A systems approach to scale up emphasises that scale up activities (i.e., activities such as obtaining and maintaining the resources and implementation capacity needed for large-scale intervention delivery), should focus on generating changes within a system to achieve the desired population level outcome [14]. For example, the characteristics of the target system(s) that scaling occurs within are considered from the outset of scale up planning, in order to identify how best to reorientate that system to achieve the desired impacts on health [14]. Given that there is huge variance in definitions for terminology used in systems science more broadly [29], for the purposes of this review, we use the definition of a systems approach to scaling up from Koorts and Rutter (2021) [14], which informs the analytical framework for data synthesis and interpretation of our findings.

### Eligibility criteria

Studies were eligible for inclusion based on the following criteria: 1) they involved interventions with a primary outcome targeting at least one of the four main behavioural risk factors for NCDs (physical inactivity, tobacco use, alcohol consumption and diet); and 2) interventions were conducted/planned for implementation in a real-world setting *with a focus* on scale up/scale up outcomes (effectiveness, scale-up, dissemination, translation or implementation studies [including randomised controlled trials *with a focus* on scale up/scale up outcomes], or protocols) or an evaluation of a previously scaled intervention (i.e., scaled up in a real-world setting). Eligible studies must have also included the term ‘system(s)’ and described either the approach/strategy taken during scale up or barriers and/or facilitators to the scale up process or outcomes. Exclusion criteria included: 1) studies testing intervention efficacy only or *without a focus* on scale up/scale up outcomes (e.g., randomised controlled trials, feasibility, and pilot studies); 2) reviews; and 3) studies applying or testing a policy (i.e., no intervention was implemented). For the purposes of this review, we defined an ‘intervention’ as “a set of actions with a coherent objective to bring about change or produce identifiable outcomes” [33], and excluded those described as a policy, strategy or government regulation.

### Information sources and search strategy

The following online databases were searched online for peer reviewed English language articles published on or after January 1st, 2016, until 31st October 2021: EBSCOHost, Medline, CINHAIL, Sportdiscus, Global Health, PsychINFO and EMBASE. This search time frame (2016–21) was chosen in response to recent calls in public health for the use of systems approaches to address complex population level problems [20], and it would enable us to capture more recent interventions that were scaled post publication of key global action plans (e.g., [2, 3] and [4]). Grey literature was searched via Google Advance and the first ten pages were screened for inclusion. The search strategy (Additional file 2) was developed and tested in consultation with a Deakin University research librarian, drawing on previous systematic reviews of related topics (e.g., [22, 34–36]), and informed by the PICO(T) (participants/population; intervention; comparator; outcomes, time) methodological approach, as recommended by Cochrane reviews [37, 38]. In this paper, *Participants/populations* were any age; the *Intervention* needed to target at least one of the four main behavioural risk factors for chronic disease (physical inactivity, tobacco use, alcohol consumption, diet); the *Comparator* was not required as this review focused on studies conducted in real-world settings; the *Outcomes* included the approaches and strategies to scaling up, barriers and facilitators experienced, and how ‘systems’ has been conceptualised and used; and the *Time* was 2016–2021.

### Study selection

Search results were imported into data management software Covidence (https://www.Covidence.org), and duplicates from the search were automatically removed. One researcher (NR) screened article titles against inclusion and exclusion criteria. All abstracts and full texts were screened by two authors independently (CS, JM). Where discrepancies in study inclusions occurred, a consensus agreement was made by four authors (CS, JM, HK and KB). Where there was incomplete information to determine scale up approaches or scale up frameworks used, reference lists and forward searching was undertaken by JM and HK.

### Quality appraisal

The Mixed Methods Appraisal Tool (MMAT) version 2018 [39] was used to appraise the quality of included studies as it encompasses multiple study designs (e.g., qualitative research, randomized controlled trials, non-randomized studies, quantitative descriptive studies, and mixed methods studies) which was reflective of the included study designs. JM and KB conducted the quality appraisal independently using the MMAT screening questions and relevant checklist questions (by each of the five study design categories) in the Covidence software. Options for each question were yes, no or can’t tell. Any disagreements in quality appraisal were resolved by discussion between JM and KB and coming to a consensus decision. As per MMAT instructions, an overall score from each question was not calculated [39].

### Data extraction

Data were extracted independently by two authors (CS, JM), with other authors (HK, KB) consulted for clarification where necessary. Data extraction included: author, year of publication, title, country or region of intervention scale-up, study design, aim, adaptions made (e.g. intervention, approach, setting), level of scale up (e.g., state/national level), intervention duration, target population, target behaviour (physical inactivity, tobacco use, alcohol consumption, diet), implementation setting, target intervention, name/number of organisations/stakeholders involved in scale up, role of organisations/stakeholders involved in scale up (e.g., funder, evaluator), how the term ‘system(s)’ has been conceptualised and used in the broader context of intervention implementation and scale up, framework/definition of scale up, method/approach to scale up, evaluation design for intervention effectiveness and evaluation of scale-up, data collected, and reported barriers and facilitators to scale up. Extracted data were tabulated (by JM and HK) to present study characteristics and results.

### Data synthesis

Following guidance on narrative synthesis methodology [40], JM created a qualitative textual description for each included study, containing information on the scale up approach described, and how the term ‘systems’ and systems approaches were conceptualised, used or informed each study. Studies were required to have included the term ‘system(s) in order to be eligible for inclusion in the review and were not required to have described or implied any use of a systems approach to scaling up. Based on the textual descriptions and in accordance with the main aims of this review, two authors (HK and KB) independently created an initial set of categories (*n* = 8 and *n* = 10, respectively) to capture how systems approaches had been used to inform and understand: (i) approaches and strategies to scaling up public health interventions; (ii) barriers and facilitators to scaling up; (iii) the evaluation of scale up processes and outcomes; and (iii) how the term ‘systems’ was conceptualised and used in each study. Initial categories were discussed and refined by HK, KB, CS and JM until consensus was reached, to produce a final set of six categories that comprised the final analytical framework for data synthesis (see Table 1 below).

**Table 1:** Characteristics of high, moderate and no use of systems approaches to scaling up, based on [14]

Analytical Framework Category	Category description	Use of systems approaches in scaling up		
		*High*	*Moderate*	*No*
1	Explicit use of systems approach in scaling up that was clearly described or defined	x		
2	Systems thinking informed intervention and implementation or scale up approaches	x		
3	Intervention approach or implementation strategies targeted different systems, or intervention targeted systems change. Systems approach not explicit or described		x	
4	Intervention multi component and may target multiple settings/sectors or system levels. Systems approach not explicit or described		x	
5	The term ‘system(s)’ included but use does not relate to a systems approach to intervention implementation or scale up			x
6	Does not adopt a systems approach to intervention implementation or scale up			x

For eligibility, studies included in the review were required to have included the term ‘system(s)’

Based on the definition of a systems approach to scaling up [14], the six categories within the analytical framework were assigned to one of three groups, according to their alignment with the definition: (Group 1; Analytical framework category 1 and 2) *High use* of systems approaches in scaling up (e.g., studies in which systems thinking informed the intervention design, implementation and scale up approach, the intervention had a focus on system changes); (Group 2; Analytical framework category 3 and 4) *Moderate use* of systems approaches in scaling up (e.g., studies in which the intervention had a focus on system changes or the role/influence of system factors, but did not explicitly adhere to the definition of a systems approach to scale up) and; (Group 3; Analytical framework category 5 and 6) *No use* of systems approaches in scaling up (e.g., studies in which the intervention may involve multiple strategies in multiple settings/sectors targeting different levels, but did not explicitly adhere to the definition of a systems approach to scale up). Table 1 presents the six categories within the analytical framework, against the three levels of a systems approach to scaling up.

Using the analytical framework (Table 1), a narrative synthesis was undertaken by JM with HK. Key findings and descriptions in each study were coded in line with the analytical framework. Themes were used to describe the approach to scale up and conceptualisation/use of the term ‘systems’ and a systems approach. Comparisons between themes were identified to present a synthesis of findings across studies, individual findings are only reported where themes were unique to an individual intervention or study. Qualitative textual descriptions relating to the barriers and facilitators to scaling up were summarised using an inductive thematic analysis approach. Descriptions were aggregated according to major themes, which informed the key barriers and facilitators reported in the Results.

## Results

**Fig. 1:**
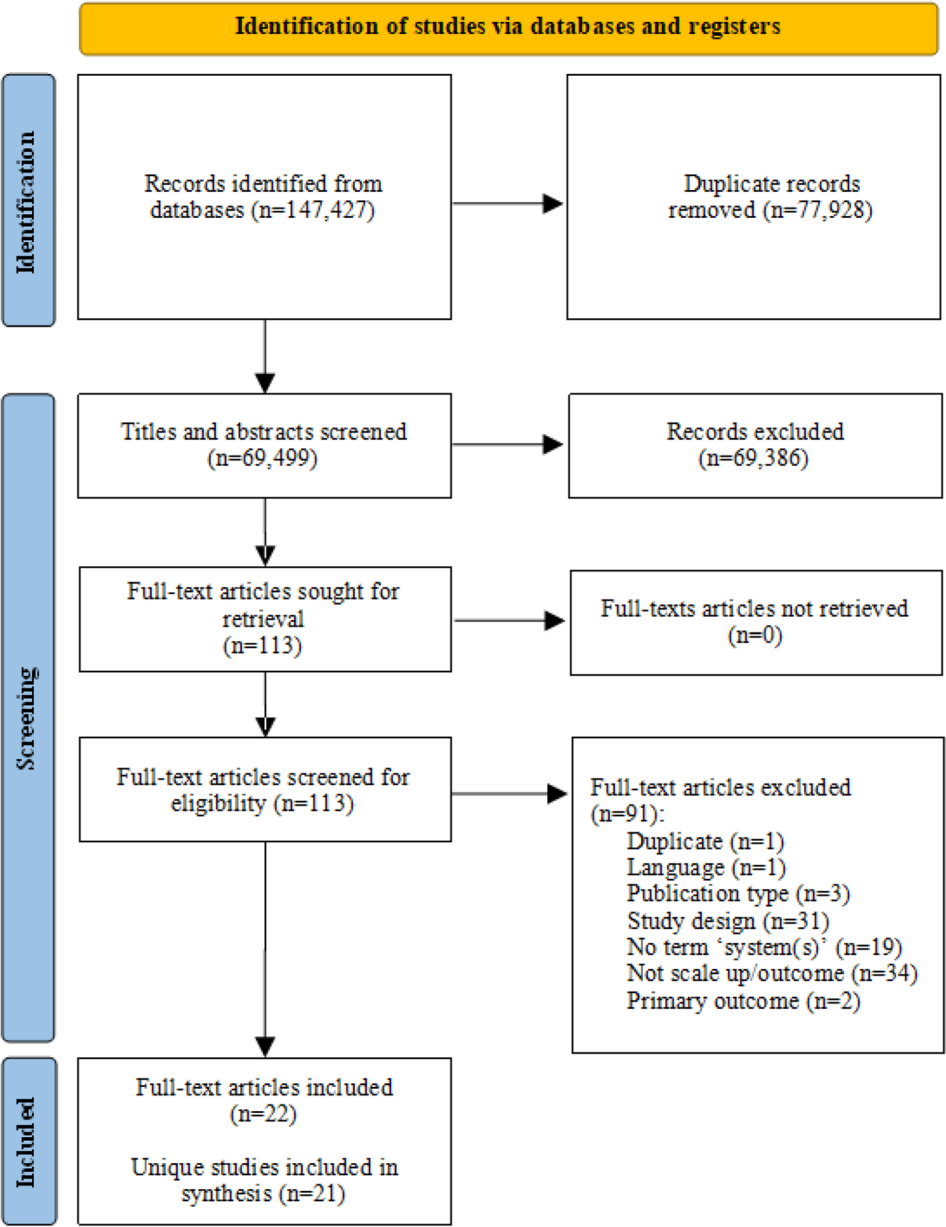
PRISMA flow diagram

The search generated 22 eligible papers, corresponding to 21 intervention studies (two papers [protocol and outcomes] addressed the same intervention). Figure 1 presents the PRISMA flowchart, which displays the number of studies screened, assessed, and included/excluded for the final review. Results reported correspond to data contained in the 22 eligible papers.

## Study sample characteristics

A description of the included papers (*n* = 22) is presented in Table 2. Of the 21 intervention studies we included in this review, 19 were discrete interventions [12, 41–60], one study involved evaluation of 16 discrete interventions [61], and one study involved evaluation of six recommended evidence-based strategies [62]. For the purposes of this review, studies that included multiple discrete programmes or strategies (e.g., [61, 62]), are reported as *one* intervention. Twenty of the 21 interventions were already being implemented at scale, only one intervention was planned for scale up in a real-world setting, with their evaluation focussing on potential scalability for future roll out [59].

**Table 2:** Study characteristics

#	Author, year, country	Title	Study design and aim	Setting, level, target population, target behaviour	Intervention overview	Scale up process	
						*Factors related to the approach/ strategies taken during scale up*	*Barriers and facilitators to scaling up*
**1**	**Berman, 2018, **[41]** USA**	Evaluation of the healthy lifestyles initiative for improving community capacity for childhood obesity prevention	Mixed method evaluation of an intervention tested at scale. The aim was to evaluate the Healthy Lifestyles Initiative	Community organisations (e.g., schools, faith-based organisations), State, children and families. Diet (for the prevention of obesity)	The Healthy Lifestyles Initiative. This included policy action, systems, environmental activities, and a messaging campaign	The ad-hoc Healthy Lifestyles Initiative conceptual model, retrospectively mapped to the Proctor framework. There were five core implementation strategies to increase uptake and penetration of coordinated policy, systems, and environmental activities in local communities and disseminate a consistent message on family healthy lifestyles The Healthy Lifestyles Initiative promoted evidence-based policy, systems, and environmental practices (7–9) more generally to support adoption and implementation across a range of community sectors, including schools, childcare providers, health care providers, businesses, non-profit community organizations, and government organizations (e.g., health departments, parks and recreation departments) Adopt new policy or change exiting policy, adopt new practices, create customised plans or goals with those served, develop or continue partnerships, initiate staff wellness activities, provide healthy lifestyles screenings or assessments, refer people served to primary care or other resources, and review organisational wellness policies	**Barriers:** Lack of guidance on the adaptations **Facilitators:** Community-capacity-building efforts (e.g., led by CDC and local hospitals and health dept.)
**2**	**Betancourt 2017, **[42]** USA**	Empowering one community at a time for policy, system, and environmental changes to impact obesity	Summary/descriptive article, which aimed to describe expansion and scale up of the Mayors Mentoring Mayors program. This was expanded to five additional states	Community level, Multi-state, Children and families. Physical activity and diet, for the prevention of obesity	A healthy communities toolkit, which helped to guide communities with best practices for health. It includes data to show how health supports the economy, tools for analysis, funding and resources, planning, and tools for city projects	A multi-state approach was used to expand the Mayors Mentoring Mayors program. Partnership was used to identify new mayors Mayors Mentoring Mayors initiative. Build capacity within local communities to reduce obesity by implementing environmental and policy changes that support healthy living. By working to improve the built environment (i.e., parks, bike lanes, sidewalks, farmer’s markets, healthy food retailers, and community gardens), government officials can lay the foundation for improved health and well-being of their residents	**Facilitators**: Involving key stakeholders in the planning and implementation GHC where the mayor was involved had the most significant changes toward better health. These mayors were recruited to share their successes, lessons learned, and best practices with their colleagues through a series of Lunch & Learns
**3**	**Blake, 2021, **[59]** Australia**	The ‘Eat Well @ IGA’ healthy supermarket randomised controlled trial: process evaluation	Mixed method process evaluation to investigate the experiences of customers, staff and stakeholders involved in Eat Well @ IGA development and implementation, maintenance and scalability	Independent Grocers of Australia (IGA) supermarket stores in regional Victoria, Australia. Supermarket customers (adults). Heathy eating	The intervention included three components: (i) signage, (ii) local area and in-store promotion; and (iii) shelf tags highlighting (healthiest) packaged foods store-wide	Co-design and strong collaboration with multisectoral partners essential for sustainability, and for planning and evaluating implementation strategies Diffusions of Innovations (Rogers 2003) underpinned evaluation, to inform on potential scalability of the intervention Stakeholders that prioritised the value on community health	**Barriers:** lack of intervention effectiveness may inhibit scale up, as well as sustainable funding and significant buy-in to ensure implementation is sustained at scale. g Organisational and resourcing difficulties were potential for scale up to other IGAs **Facilitators:** Collaboration between food retailers, governments and academics Co-design approach to implementation planning and evaluation. Awareness of contextual factors related to the food industry on implementation and aligning stakeholder objectives and outcomes
**4**	**Bolton, 2017, **[43]** Australia**	The outcomes of health-promoting communities: being active and eating well initiative—a community-based obesity prevention intervention in Victoria, Australia	Mixed method and multi-level quasi-experimental evaluation to assess the impact of the Health-Promoting Communities: Being Active Eating Well initiative	Community settings including schools and workplaces, State, Primary and secondary school aged children, Diet and physical activity, for the prevention of obesity	The main intervention components included stakeholder engagement and partnership development, needs assessment and capacity building, social marketing	State government funding was provided to deliver activity and eating well community programs in areas of disadvantage. The intervention was based on the Colac Be Active Eat Well programme. Analysis Grid for Environments Linked to Obesity (ANGELO) framework, with input from community and stakeholder workshops, expert advisory group, steering committees	**Barriers:** insufficient implementation, time constraints, competing priorities, lack and engagement, project delays, heterogeneity in community contexts
**5**	**Conte, 2017, **[44]** Australia**	Dynamics behind the scale up of evidence-based obesity prevention: protocol for a multi-site case study of an electronic implementation monitoring system in health promotion practice	Mixed methods multi-site case studies using ethnographic design. This study examined how Population Health Information Management System (PHIMS) intersects with health promotion practice. It aimed to determine whether e-monitoring systems improve the dissemination, adoption, and ongoing delivery of evidence-based preventive programs	Local health districts, State, Childcare and elementary school aged children. Physical activity and diet, for the prevention of obesity	An E-monitoring system to facilitate the dissemination of evidence-based obesity prevention programs targeting healthy eating and physical activity practices (Live Life Well at School and Munch & Move) into every primary school and childcare centre across the state of New South Wales	PHIMS was created to support the dissemination of obesity prevention programs and is delivered across all local health districts in the state of New South Wales. This was designed, implemented, and funded by state level organisations	No information reported in paper
**6**	^a^**Davis, 2017, **[62]** USA**	Research to practice: implementing physical activity recommendations	Mixed methods evaluation describing a model for, and aims to identify important factors in, the process of dissemination and implementation of evidence-based recommendations in rural and under-resourced communities	Rural and under resourced tri-ethnic community, Rural/ regional, Community members, Physical activity	The intervention included six strategies: community-wide campaigns, creation of or enhanced access to places for physical activity, information outreach, community-scale and street-scale design, land-use policies, social support, and individually adapted health behaviour change strategies	Alliance partners studied translation of pre-existing community prevention taskforce recommendations. Alliance members then selected strategies to implement that were considered feasible in the local context The strategies were selected and implemented in ways that the Alliance thought would be feasible	**Facilitators:** Building on a community-academic partnership; engaging multiple local and external partners; employing culturally appropriate strategies; and using approaches that fit local context and place characteristics (topography, land ownership, population clusters, and existing roadways)
**7**	**Fernandez, 2016, **[45]** Canada**	Factors influencing the adoption of a healthy eating campaign by federal cross-sector partners: a qualitative study	A qualitative study that aimed to describe factors that influence cross-sector partner decisions to adopt the Eat Well campaign	Government, council, and community, National, Children. Diet, for the prevention of obesity	The Eat Well Campaign is a social marketing campaign that focusses on food skills	Cross-sector partners, including food retail, advertising, media, government, and non-government organisations adopted components of the Eat Well Campaign. Adoption was influenced by values, attitudes, and the notion of partnership Organisations from different industry and sectors (e.g., food retailers, NGOs, advertising groups) that have different means of influencing the system	**Barriers:** Lack of resources and capacity; reserved/conservative attitudes towards the intervention and the lack of exposure to the mass media channels; political issues; strict control of information by the intervention; lack of relevant population groups; lack of clear objectives; difficulties integrating activities within the organisation **Facilitators:** Targeted approach selecting partners through networks with high opinion leadership value (invitation-based approach); high perceptions of fit and favourable attitudes; feel-good nature of the campaign; recognizing in-kind and paid collaborators; previous experience and relationships; desire to be a part of a group; simplicity/ complexity of the innovation; having skills and knowledge
**8**	**Gelli, 2016, **[63]** Ghana**	Evaluation of alternative school feeding models on nutrition, education, agriculture, and other social outcomes in Ghana: rationale, randomised design, and baseline data	Protocol for a cluster randomised trial to evaluated the impact of a large-scale school meals program in Ghana on school-age children’s anthropometry	Schools, caterers, and farmers, National, school aged children, diet, and malnutrition	The Government of Ghana School Feeding Program (GSFP), which is a school catering intervention	The intervention/ program was implemented across 10 regions of Ghana. The program was government funded and overseen by a ministry department with additional support from a school implementing committee. Caterers are provided funding to purchase food from markets Multiple sectors to increase food production, household income, and food security in deprived communities	**Barriers:** Delays in disbursement; suboptimal service delivery; older children progressing to secondary school less likely to receive intervention Implementation requires multiple stakeholders, in Ghana are challenges with information flow, supervision and monitoring between these different stakeholders **Facilitators**: Ability of communities to actively engage in program and the strengthening of public institutions involved
	**Gelli, 2019, **[46]** Ghana**	A school meals program implemented at scale in Ghana increases height-for-age during mid childhood in girls and in children from poor households: A cluster randomised trial	Outcome evaluation of a cluster randomised trial that evaluated the impact of a large-scale school meals program in Ghana on school-age children’s anthropometry	Schools, caterers, and farmers, National, School aged children, diet and malnutrition	The Government of Ghana School Feeding Program (GSFP), which is a school catering intervention		
**9**	**Hassani, 2020, **[47]** Canada**	Implementing appetite to play at scale in British Columbia: Evaluation of a capacity-building intervention to promote physical activity in the early years	A mixed methods study that examined the implementation and impact of Appetite to Play scale-up using the RE-AIM framework (Glasgow et al. 1999)	Community childcare providers, State, Early years children and educators, physical activity, and healthy eating	Appetite to Play is a capacity building intervention for childcare providers. Capacity building was achieved via training, toolkits, technical support, community of practice, and marketing and communications	The scale-up and implementation of Appetite to Play was based on consultation and engagement with early years stakeholders across the province. A “train the trainer” model was used to spread the capacity-building intervention across the province. Master trainers trained regional trainers. Regional trainers then delivered workshops within the community. Training was embedded into existing training infrastructure A provincial stakeholder advisory group was set up to advise the partnership group on the development of both the resource, scale-up strategy, implementation (course correction), and to create a framework for sustainability	**Barriers:** Environment (space, weather); multiple initiatives occurred provincially **Facilitators:** Organisational supports (equipment and indoor space); resources (planning, access to websites/books); mandate (childcare facilities were required to adhere to PA policies); stakeholder network engagement
**10**	**Hunt, 2020, **[58]** UK**	Scale-Up and Scale-Out of a Gender-Sensitized Weight Management and Healthy Living Program Delivered to Overweight Men via Professional Sports Clubs: The Wider Implementation of Football Fans in Training (FFIT)	Summary/descriptive article describing the development, evaluation and scale up of FFIT, mapped onto the PRACTIS guide (Koorts et al. 2018), including scale up outcomes and adaptations to scale out into other contexts	Implemented via professional football clubs in Scotland and England, UK, targeting overweight/obese men aged 35–65 years, with a BMI > 28kgm^2^. Targets healthy diet and physical activity for the prevention of obesity	FFIT is a 12-week group-based weight management and healthy living program. Implemented by trained club coaches	Implementation of FFIT builds on and develops existing structures within clubs; congruence with aims and aspirations of newly-established Scottish Premier League Trust (SPL-T); congruence with public health priorities to address rising obesity, poor diet and physical inactivity. Funding secured from the Scottish Government to reimburse clubs Uses infrastructure & experience of FFIT & other community football initiatives as an overarching organisational structure, supporting community coaches within clubs to deliver health-promoting programs to adults The PRACTIS guide (Koorts et al. 2018) was retrospectively applied to describe intervention development, evaluation and scale up	**Barriers:** Coaches lack of experience in target population, scepticism, competing demands on coaches time and club facilities. Lack of a fully developed model for routine scale up, prior to licensing model **Facilitators:** Intervention effectiveness and cost effectiveness. Cultural push and pull factors. Organisational/provider level included adequate resources, coaches and community wing, and funding. Organisational buy-in and support, use of existing infrastructure and skills of coaches. Established reputation
**11**	**Joyce, 2018, **[48]** Australia**	The ‘Practice Entrepreneur’ – An Australian case study of a systems thinking inspired health promotion initiative	A qualitative case study that examined the experience of practitioners involved in a state-wide systems intervention	Healthy Together Victoria (HTV) included settings-based initiatives (i.e., the Achievement Program), community based programs, social marketing strategies, and research/policy initiatives. Community members across all aged. Broadly, HTV targeted priority areas of physical activity, tobacco use, alcohol consumption and diet; obesity prevention becoming the dominant focus over time	HTV was a systems initiative that included several policy and program initiatives used in combination. This paper focussed on one of HTV settings initiative: the Achievement Program that targeted schools, workplaces, and early childhood services	Healthy Together Victoria was developed by the State Government. Fourteen local governments receive funding to implement the initiative, with most of the funding directed towards staffing Explored how system elements were connected, how organisations were connected (e.g., schools, workplaces, early childhood services, and local health and welfare organisations), and understanding of the values and connections between organisations, people, policies, and programmes	**Barriers:** Harder process related to planning and getting approval to implement identified opportunities within local government; lack of understanding of systems and rigid approaches to planning in partner organisations **Facilitators:** Agile in planning and implementation; access to multiple departments within local government; Easier to implement a systems approach in smaller, finite, well-defined communities; local adaptation and reflective process, more opportunities to collaborate
**12**	**Livingston, 2020, **[49]** USA**	Reducing tobacco use in Oregon through multisector collaboration: aligning Medicaid and public health programs	Summary/descriptive article describing an implementation evaluation. It aimed to evaluate the impact of the state-wide smoking cessation intervention	Coordinated care organisations (CCOs), State, Medicaid members, Smoking and tobacco use	A state-wide CCO cigarette smoking incentive metric for Medicaid members in 2016. The intervention included a cross-agency collaboration to decrease smoking via increasing smoke-free environments, increasing public awareness about risk, and supporting access to cessation services through Quitline. It was combined with tobacco surveillance. A tobacco incentive metric to encouraged reductions in tobacco prevalence	These healthcare strategies were first established via collaboration between public health and healthcare partners. Accountability and incentives support continued partnership and application Multifaceted cross-agency approach that includes improving access and affordability to cessation services, ensuring the places people live, work, play, and learn are tobacco-free and reinforce individuals desire to quit or never start Working together to ensure alignment across state-level programs, policies, and systems and to support CCOs in their efforts to reduce tobacco prevalence	**Facilitators:** Cross-agency alignment. Multisector intervention statements. Connecting local public health departments and CCOs
**13**	**Lonsdale, 2016, **[50]** Australia**	Scaling-up an efficacious school-based physical activity intervention: Study protocol for the ‘Internet-based Professional Learning to help teachers support Activity in Youth’ (iPLAY) cluster randomized controlled trial and scale-up implementation evaluation	A protocol for a cluster randomized controlled trial and scale up implementation study. The aim was to evaluate the intervention’s effectiveness and implementation at scale	Schools, State, Primary school aged children, physical activity and fitness	iPLAY is a multi-component intervention supported by mentors and online learning platform	All state funded schools in NSW will be invited to participate in scale up of the SCORES intervention. The scale-up implementation study will be guided by the RE-AIM framework	No information reported in the paper
**14**	**Malakellis, 2017, **[51]** Australia**	School-based systems change for obesity prevention in adolescents: outcomes of the Australian Capital Territory ‘It’s Your Move!’	A quasi experimental, repeated measures study. The purpose was to utilise a systems intervention to prevent obesity development in children and to test the practical application of a systems approach	Schools, State, Adolescents/ secondary school aged children. Diet for the prevention of obesity	A food at school initiative, which encompasses the whole food system. Includes canteen food, food at fundraising, and events. This was achieved via collaboration with local food producers and nutrition Australia to develop policies, commit to increasing healthy food consumption among staff and students, a focus on the relationship between health and food across all areas of the curriculum, traffic light colour coding of food sold at the canteen, provision of healthy foods and reduction of unhealthy foods at events, healthy morning teas for staff to encourage role modelling, cooking classes outside school hours for students and families, and increased access to water fountains	The intervention was an extension of an ‘It’s your move’ program implemented previously in Victoria. The program was adopted to incorporate a greater systems approach. Government schools in the Australian Capital Territory were provided funds to support participation	**Barriers:** Time and resources are needed to detect systems changes. Allow for sufficient lead in time
**15**	**Matheson, 2019, **[12]** New Zealand**	Strengthening prevention in communities through systems change lessons from the evaluation of Healthy Families NZ	A mixed method comparative case study. This study aimed to evaluate the Healthy Families New Zealand initiative	Rural and urban communities with high levels of socioeconomic deprivation, National, Community members, all four chronic disease risk factors (diet, physical activity, tobacco use, alcohol consumption)	Investment in dedicated systems thinking and acting health promotion workforce and activating local leadership to influence change	Healthy Families NZ is a government funded initiative delivered in areas with higher socio-economic deprivation. The initiative is delivered via partnership with indigenous organisations, regional sports trusts, and local councils	**Facilitators:** Quality leadership; highly skilled and flexible workforce to meet local needs and adapt; stability in workforce and Strategic Leadership Groups (SLGs); early demonstration of implementation success; sensitivity to initial conditions; being able to develop deep connections into diverse communities; facilitating strategic alignment between organizational and community leaders; local government connections **Barriers:** Remote and dispersed geographical location; recruitment, stability, and cohesion of the workforce; staff turnover; time spent on understanding how to implement work; modest resources; siloed and competitive service funding approaches; competing interests of industries; lack of data at the local level
**16**	**McKay 2021, **[52]** Canada**	Status Quo or Drop-Off: Do Older Adults Maintain Benefits from Choose to Move-A Scaled-Up Physical Activity Program-12 Months After Withdrawing the Intervention?	A type 2 hybrid effectiveness implementation study design that evaluated the maintenance of intervention gains 12 months following delivery of the Choose to Move intervention	Community, State, Older adults, Physical activity	The Choose to Move intervention is not prescriptive but was designed as an adaptable model whereby participants choose what they enjoy and are able to do. At the organization level it builds community capacity to support awareness of, and access to, local health-promoting opportunities. At the individual level, it provides personalized support to create action plans customised to individual’s activity preferences, resources, and mobility capacity	The implementation approach adopted core element of frameworks that consider research to practice, community-centred implementation, multi-level stakeholder perspectives, and two-way communication. Scale up was facilitated via partnership that included government and community organisations An integrated approach to scale-up acknowledges levels of influence on behaviour change across a socioecological continuum that spans individual to systems-level influences	**Facilitators:** Participants’ positive relationship with the activity coach; participants’ interactions with other participants
**17**	**Nettlefold, 2021, **[53]** Canada**	Scaling up Action Schools! BC: How Does Voltage Drop at Scale Affect Student Level Outcomes? A Cluster Randomized Controlled Trial	A cluster randomised controlled trial. This study aimed to describe strategies to support implementation and scale-up, evaluate implementation and students’ PA/ fitness, assess relationships between teacher-level implementation and student outcome	Schools, Province wide, Children, Physical activity	Action Schools! BC is a whole of school model that provides elementary schools and generalist teachers with tools and support to create physical activity action plans	Implementation and scale up was led by a support team. Strategies included engaging and consulting with interested schools, funding and providing teacher training, resource provision, incentives, development of advisory committees, and mentorship with ongoing consultation A whole of school model that provided elementary schools and generalist teachers with tools and support to create PA action plans. Recognising schools are complex and dynamic systems	**Barriers:** Schools were acknowledged to be complex and dynamic systems, posed challenges to scaling up **Facilitators:** Governance, leadership, resources, outsourcing delivery, accountability structures and committed stakeholder engagement. Capacity-building activities. Multi-component model with flexibility that allowed schools' autonomy. New implementation strategies were added over time
**18**	^a^**Rechis, 2021, **[61]** USA**	Be Well Communities: mobilizing communities to promote wellness and stop cancer before it starts	Mixed method evaluation of 16 different interventions implemented via six collaborating organisations	Schools, food banks, YMCA, childcare settings, community settings and tourism settings. Community members including children, families, and adults. Tobacco use, healthy eating, physical activity, UV exposure, inadequate preventive care (cancer screening and human papillomavirus vaccination) **Note**: tobacco control and HPV vaccination elements of Be Well Communities reported as underway, not evaluated in included paper	The multiple interventions included healthy food initiatives, school-based Physical Education, an education program, active classrooms, extracurricular activities, places for physical activity, community physical activity, skin cancer prevention intervention, outdoor settings, and childcare/school interventions	Utilised partnership and followed a collective impact model. Stages included community assessment, planning, implementation, and then sustainability The steering Committee built a deep collaborative network. The initiative was led by 'wants and needs of the community' that establishes the infrastructure, prioritises target areas and community action plan	**Facilitators:** Deep collaborative network **Barriers:** Natural disasters
**19**	**Tong, 2020, **[56]** USA**	The Emergence of a Sustainable Tobacco Treatment Program across the Cancer Care Continuum: A Systems Approach for Implementation at the University of California Davis Comprehensive Cancer Centre	Summary/descriptive article describing use of an implementation framework to describe the emergence of a sustainable tobacco treatment program across cancer care, using a systems approach	A comprehensive cancer centre, State, Smokers, Smoking	The sustainable tobacco treatment program. The intervention targets multiple points across the cancer care continuum	Utilised a Consolidated Framework for Implementation Research. This covers components of the intervention, individuals and stakeholders, the outer setting/ context, the inner setting or context, and the implementation process The study applied constructs from an implementation research framework to describe implementation of the programme	**Barriers**: Leadership engagement (e.g., coordination, reporting pathways), resources (money, training/ education, physical space, time), access to information and knowledge **Facilitators**: Core components of the intervention: referral to Quitline or UCD Group Class. Quality programmes and accreditation standards that support operations. Leadership engagement. available resources, knowledge, and information
**20**	**Wilcox, 2018, **[57]** USA**	Faith, Activity, and Nutrition Randomized Dissemination and Implementation Study: Countywide Adoption, Reach, and Effectiveness	A group randomised trial. The study aimed to report the countywide adoption, reach, and effectiveness from the Faith, Activity, and Nutrition dissemination and implementation study	Community church organisations, County level, Church members, healthy eating and physical activity	Church committee training provided an overview of physical activity and healthy eating guidelines and benefits. A set of activities, handouts, messaging, educational materials, bulletin board, would suggest policies that a pastor could set. Regular technical assistance provided	Churches and faith-based groups across the county were invited to participate. Adaptations to the original intervention were made to support broader dissemination. Community health advisors deliver training to churches and each church group formed a committee that received training, funding, held meetings, and implemented the program with their church. Implementation evaluation was guided by the RE-AIM framework The structural model of health behaviour by Cohen et al. guided the intervention (availability of protective or harmful products, physical structures (or physical characteristics of products), social structures and policies, and media and cultural messages) Church environment, church policies, church systems, rather than individual church members. Use of community members/ 'lay leaders'	**Facilitators:** Earlier participation in initiatives led to improved participation. Building relationships, having a community presence, and making multiple contacts are important ingredients for engaging churches in health promotion efforts
**21**	**Wolfenden, 2020, **[60]** Australia**	From demonstration project to changes in health systems for child obesity prevention: the legacy of ‘Good for Kids, Good for Life’	Summary/descriptive article from key policy and practice stakeholders describing the Good for Kids, Good for Life (GfK) initiative and its impact	Whole-of-community initiative, using a community settings approach targeting childcare services, schools, community service organisations, sporting clubs, health services and Aboriginal communities. Targeting physical activity and diet (for the prevention of obesity)	Initiatives targeted childhood obesity via healthy eating and physical activity policies and practices. Initiatives delivered in each setting were prioritised using a public health planning framework informed by local stakeholder input, Aboriginal cultural review and expert review of published evidence	Implementation strategies used in the service delivery models to enhance consistent implementation of the initiatives at-scale in and across the settings included soliciting organisational leadership, provision of program resources and information, training workshops, follow-up support, accreditation schemes and feedback Implementation was facilitated through a surveillance system to assess implementation of targeted policies and practices. Feedback was provided on performance targets, evidence to inform program planning, monitor program achievements and enable provision of feedback to community organisations	**Facilitators**: Surveillance system to capture implementation data routinely. Increased capacity of health service staff. Significant state and national funding for prevention and alignment with a policy need Engagement of end-users and local and state-level leadership

*BC* British Columbia, *USA* United States of America, *NZ* New Zealand, *UK* United Kingdom, *UV* Ultraviolet, *PHIMS* Population health intervention management system, *REAIM* Reach, Effectiveness, Adoption, Implementation and Maintenance framework (Glasgow et al. 1999); *PRACTIS* PRACTical planning for Implementation and Scale up Guide (Koorts et al., 2018)

Column 2 ‘country’ corresponds to country of intervention implementation in included paper

^#^ Corresponds to intervention number, not paper number

^a^Indicates study counted as ‘one’ intervention but involved multiple discrete programs or strategies

According to World Bank categorisations [64], the 21 interventions were implemented in five high income countries (United States of America [41, 42, 49, 56, 57, 61, 62], Australia [43, 44, 48, 50, 51, 59, 60], Canada [45, 47, 52, 53], New Zealand [12] and the UK [58]), and one low income country (Ghana [46, 63]).

Seven interventions targeted physical activity and diet [27, 42, 44, 47, 57, 58, 60], four targeted diet [41, 45, 51, 59], four targeted physical activity [50, 52, 53, 62], two targeted tobacco use [49, 56], two targeted all four behaviour risk factors (physical inactivity, tobacco use, alcohol consumption and diet) [12, 48], and one targeted diet and malnutrition [46]. One study targeted physical activity, tobacco use, diet, UV exposure and preventive care (e.g., cancer screening and human papillomavirus vaccination) [61], but the tobacco control and HPV vaccine elements of the intervention were reported as under way and were not included by the authors as part of their evaluation [61].

Interventions targeted health improvements or behaviour change among children/adolescents and families [41–44, 46, 47, 50, 51, 53, 55, 60, 61, 63], older adults [52], smokers [49, 56], men [58], and general community members, including community based partner organisations/practitioners (e.g., food banks and the YMCA) [12, 45, 47–49, 57, 59, 61, 62]. Interventions targeted multiple community-based settings, such as schools [41, 43, 44, 46, 50, 51, 53, 61, 63], childcare settings [44, 47, 61], faith-based organisations [41, 57], local health districts and local governments [44, 48], workplaces [43], professional football clubs [58], supermarkets [59], coordinated care organisations [49], cancer centres [56], and in the community at a broader environmental/policy level [12, 42, 45, 48, 49, 52, 60–62].

Of the 22 papers reviewed, there was one qualitative study [45], one type 2 hybrid effectiveness implementation trial [52], one group randomised trial [57], two quasi-experimental designs [43, 51], three case studies [12, 44, 48], four cluster randomised controlled trials [46, 50, 53, 63], five mixed method evaluations [41, 47, 59, 61, 62], and five summary/descriptive articles describing intervention and scale up processes/outcomes generally [42, 49, 56, 58, 60].

### Approaches and strategies to scaling up

There were varying approaches and strategies used to reach at-scale implementation (Table 2). Fifteen (71%) of the 21 interventions were described as ‘designed for scale’. These interventions were developed based on prior evidence from other programmes, and scaling required strong research-practice partnerships and existing resources customised for stakeholders. Of the 15 interventions designed for scale, 10 (67%) were implemented without the reported need for small scale pilot trials, and were scaled across a single community [62], multiple communities within one state [48, 60, 61], across a whole state/province [41, 44, 47], across multiple states [42], or nationally [12, 45]. Five (33%) of the 15 interventions followed a traditional translation pathway of a phased approach from small-scale controlled efficacy testing to plan for scale up [59], and from small-scale controlled efficacy/feasibility testing to then effectiveness and implementation testing at a state [50, 57] and province level [52, 53].

Four interventions were expanded due to earlier effectiveness outcomes, and ongoing government funding and support. Two of these three interventions started as smaller community projects that were incrementally expanded to reach multiple communities or settings state-wide [43, 51], and two began as pilot trials that were incrementally expanded to reach nationally [46, 58]. Two interventions targeting tobacco reduction were scaled as part of existing health system infrastructure. One was the extension of more than two decades of established state level policies and evidence-based initiatives [49], and the other was integrated as part of an existing large, comprehensive health system [56].

### Barriers and facilitators to scaling up

Forty-one textual descriptions relating to barriers and facilitators to scaling up were summarised from nineteen included studies (Table 2). Six themes emerged, which included four facilitators (committed stakeholder engagement, capacity building in the local workforce, flexibility in delivery and implementation, and ongoing programme feedback and improvement) and two barriers (competing interests and priorities, and insufficient time and resources).

#### Facilitator 1: Committed stakeholder engagement

Full engagement of stakeholders was key for scale-up success, as reported in several studies [12, 41–43, 45, 47–49, 53, 56–60, 62]. Early involvement and participation of stakeholders in the planning and implementation of initiatives were also identified as important [12, 57, 58]. End users and leadership were the most mentioned stakeholders/aspects that played a key role in the scale up process. A weight management programme targeting men with overweight or obesity leveraged the popularity of professional football among target users, and existing organisational structure of local football clubs, as delivery systems to provide a sustainable model for wider implementation of the programme [58]. Several studies reported that the initiatives, and implementation process, were driven by the local contexts and needs and resulted in scale-up successes [12, 41, 43, 48, 62]. Strategically engaging leadership and facilitating alignment in goals and missions across different organisations and communities was associated with a greater degree of collective actions [12, 45, 56, 59, 60].

#### Facilitator 2: Capacity building in local workforce

Efforts in capacity building [12, 41, 53, 57, 58] were common in the included scale-up initiatives. Capacity building for the implementation workforce was necessary, especially in systems thinking guided initiatives. This could include providing adequate resources and funding to support existing infrastructures. Understanding and responding to the adaptive nature of systems were recognised as a key skillset for local workforce [12, 48].

#### Facilitator 3: Flexibility in delivery and implementation

Initiatives that had a multi-component or flexible delivery model were more acceptable to local implementation as they allow for autonomy [12, 53]. The flexibility also enabled initiatives to be appropriately adapted to fit local context and place characteristics [58, 62]. By contrast, rigid and controlled approaches in the initiatives appeared to hinder implementation [45, 48].

#### Facilitator 4: Ongoing programme feedback and improvement

Establishing and utilising an ongoing evaluation and feedback system was perceived instrumental in scale up [57, 59, 60]. In a whole-of-community child obesity prevention programme, ongoing monitoring and feedback of programme achievements to community organisations informed quality improvement and innovation, which resulted in a significant increase in the promotion of community settings implementing the evidence-based practice [60].

#### Barrier 1: Competing interests and priorities

Implementation of initiatives was challenged by conservative/risk averse attitudes of local organisations [45], competing priorities of local organisations [43], and competing interests of industries [12, 59]. For example, organisations were reluctant to adopt the initiative due to a low perception of risk versus benefits, i.e., potential low relative advantage [45]. Another healthy supermarket intervention described the challenges in promoting sales of healthy foods due to perceived customer demand and supplier contract agreements [59].

#### Barrier 2: Insufficient time and resources

Lack of appropriate resources such as funding was identified as a barrier to successful scale-up [12, 45, 53, 55, 59]. For one intervention that targeted different points of the system [12], siloed and competitive funding approaches in the government impacted the financial security of the initiative.

### Use of systems approaches to scaling up

Tables 3, 4 and 5 summarise how studies categorised in the high, moderate and no use of a systems approach groups, respectively, articulated how ‘systems’ was conceptualised or used, in what ways a systems approach was adopted, and the methods/theoretical frameworks or principles applied to study scale up processes or outcomes.

**Table 3:** Studies categorised as ‘high use of a systems approach’ (*n* = 5)

Author, year	Target behaviour	Conceptualisation and use of ‘systems’ and a systems approach	Methods, theoretical frameworks and/or principles adopted to study scale up processes or outcomes
**Conte et al. (2017)** [44]	Physical activity and diet (for the prevention of obesity)	Recognised health care settings as complex adaptive systems and that they should act consistently with this theory for quality improvement purposes. Recognised that practice occurs as part of a social system, and practitioners have agency and multiple accountabilities within the system(s). System also referred to in context of ‘monitoring systems’ for implementation of health promotion	No framework to guide scaling up reported in paper. Social network analysis was used to quantify how connected practitioners were with each other, how central/or isolated some players were, and why it was easier to diffuse information across some groups
**Joyce et al. (2018)** [48]	Physical activity, tobacco use, alcohol consumption and diet (for the prevention of obesity)	Systems thinking used to inform implementation. Systems science described as a broad class of analytical approaches that aim to uncover the behaviour of complex systems. Applied systems thinking practice, systems mapping to draw connections with staff from other stakeholder organisations and discuss common concerns, look for common interests and potential strategies to align as many community groups as possible, decide on which issues to prioritise	Systems thinking allowed practitioners to act as ‘practice entrepreneurs’, a concept developed in the paper to describe the more reactive and flexible approach in practice. Systems mapping was used to explore common practice, concerns, interests, and values of other organisations. Identified interrelationships between key attributes of the system (such as programs, practitioners, networks and organisations), and leverage points that could produce action across these multiple actors. Engaging key organisations and people linking them was a key indicator of success. Outcomes included levels of population reach and engagement
**Malakellis et al. (2017)** [51]	Diet (for the prevention of obesity)	Systems thinking informed implementation and the intervention, and the intervention targeted different systems points across the whole food system	Analysis Grid for Element Linked to Obesity (ANGELO) (Swinburn et al. 1999) workshop, which includes WHO systems building blocks. Schools leveraged existing health-promoting activities and introduced initiatives informed by ANGELO. Systems mapping identified and prioritised the key determinants while considering gaps in knowledge, capacity, needs and existing health promotion initiative
**Matheson et al. (2019)**	Physical activity, tobacco use, alcohol consumption and diet	This study targeted different points in the system, used systems thinking to inform implementation, and focussed on system change. It distinguished systems thinking from linear, siloed approaches Context- and complexity-oriented. For example, interventions are described as ‘events in a system’ and need to be able to adapt to the specific social, economic, cultural, and geographic circumstances of a community	Through Strategic Leadership Groups (SLGs), guided by the WHO’s building blocks for a strong health system (WHO, 2007). A dedicated systems thinking and acting health promotion workforce and activating local leadership to influence change The study developed criteria for two outcomes of systems change: First, Prevention Infrastructure, requiring evidence of an increase in local organisations focusing on prevention and healthier practices, including through policy changes, changes in the built environment and additional resources dedicated to prevention. Second, Prevention Attitudes and Paradigm, requiring evidence of an increased commitment to prevention or seeking out opportunities to collaborate with other organizations for the purpose of prevention
**Tong et al. (2020)** [56]	Tobacco use	This study targeted different systems points, and systems thinking was used to inform implementation	The systems framework (the Cancer Care Continuum) was used to facilitate viewing plans, progress, and priorities. It helps identify research gaps, and where collaboration with others is needed to have an impact, including where more resources may be needed. The Consolidated Framework for Implementation Research (CFIR) (Damschroder et al. 2009) was used to describe and evaluate implementation

**Table 4:** Studies categorised as ‘moderate use of a systems approach’ (*n* = 7)

Author, year	Target behaviour	Conceptualisation and use of ‘systems’ and a systems approach	Methods, theoretical frameworks and/or principles adopted to study scale up processes or outcomes
**Berman et al. (2018)** [41]	Diet (for the prevention of obesity)	Intervention targeted different systems points. This included targeting organisations from different levels that influenced behaviour and utilisation of an intervention that featured a range of policy, systems and environmental strategies	The types of policy, systems, and environmental activities were broad, but activities generally were in eight categories: adopt new policy or change exiting policy, adopt new practices, create customized plans or goals with those served, develop or continue partnerships, initiate staff wellness activities, provide healthy lifestyles screenings or assessments, refer people served to primary care or other resources, and review organizational wellness policies. Initiative mapped to Proctor et al. 2013 recommendations for informing implementation strategies, no framework for guiding scale up reported
**Betancourt et al. (2017)** [42]	Physical activity, and diet (for the prevention of obesity)	This intervention focussed on system changes. It targeted policy, system, and environmental changes	Principles included long-term, sustainable policy, system and environmental changes in the community where people work, play, worship, learn and live (i.e., social determinants of health)
**Bolton et al. (2017)** [43]	Physical activity and diet (for the prevention of obesity)	Not specific/ explicit to systems but a multi-component approach targeted multiple systems levels. The strategies implemented were directed towards increasing community capacity, creating supportive environments, and promoting healthy behaviours	Analysis Grid for Element Linked to Obesity (ANGELO) (Swinburn et al. 1999) used to develop project objectives, no framework to guide scale up Principles included achieving pre-defined objectives in each community (e.g., increasing community capacity to promote healthy eating and physical activity)
**Livingston et al. (2020)** [49]	Tobacco use	The approach and strategies targeted different points in the system. The multifaceted approach included improving access and affordability to cessation services, ensuring the places people live, work, play, and learn are tobacco-free, and reinforcing individuals desire to quit (or never start) smoking	Acknowledged the policy and system infrastructure alignment required to support the state-wide Coordinated Care Organization
**McKay et al. (2021)** [52]	Physical activity	Systems not explicitly defined, however, the multi-component and multi-level intervention sought to address factors that support physical activity decisions and engagement. Acknowledged that program investment and utilising health promoting opportunities in local environments were associated with sustainment, which is linked to existing systems and services	Principles included community capacity to support awareness of, and access to, local health-promoting opportunities. No information reported in paper on scale up frameworks; ^a^scale up guided by conceptual models from Yamey (2011) and Simmons and Shiffman (2007)
**Nettlefold et al. (2021)** [53]	Physical activity	Schools were acknowledged to be complex and dynamic systems. This presented challenges to scaling up. Systems thinking was incorporated into the intervention by enabling schools and teachers to provide more physical activity opportunities for children via six action zones—school environment, scheduled physical education, classroom action, family, community, extra-curricular, and school spirit	Scale up approach drew on Simmons and Shiffman (2007) framework recommendations. Key scale up strategy included building partnerships with organisations across health, education, sport, government and community sectors
**Wilcox et al. (2018)** [57]	Physical activity and diet	Multicomponent intervention targeted different systems points within faith-based organisations. Acknowledged the role and impact of systems in a systems thinking way	Evaluation was guided by the REAIM framework (Glasgow et al. 1999). No information on scale up framework reported in paper

^a^Information sourced from reference searching when descriptions in the included paper were unclear

**Table 5:** Studies categorised as ‘no use of a systems approach’ (*n* = 9)

Author, year	Target behaviour	Conceptualisation and use of ‘systems’ and a systems approach	Methods, theoretical frameworks and/or principles adopted to study scale up processes or outcomes
**Blake et al. (2021)** [59]	Diet	System referred to in context of the broader ‘social system’ that influenced the spread of interventions. Study involved a strong collaboration between stakeholders across the system; food retailers, local government and researchers, to test intervention and potential scalability	No information on a scale up framework reported in the paper. Rogers’ Diffusion of Innovations (Rogers 2003) and the REAIM framework (Glasgow et al. 1999) guided evaluation
**Davis et al. (2017)** [62]	Physical activity	Systems referred to in context of ‘evaluation systems’, systems approach not described or utilised, but this multi-component intervention targeted multiple systems points. This included strategies in the community, policies and social support. Strong community-academic partnerships and acknowledgement of local resources, infrastructure, political climate and advocacy were essential	A community-generated logic model was used as a dissemination tool and framework for implementing strategies. Focus included committed community communication, creating and enhancing access to places for physical activity, implementing community design and land-use policies, social support, and individually adapted health behaviour change
**Fernandez et al. (2016)** [45]	Diet (for the prevention of obesity)	Systems approach not described or utilised, however, refers to ‘innovation-systems fit’ and the ‘social system’. Study utilised organisations from different industry, sectors, and system levels (e.g., food retailers, advertising groups, Federal, provincial and territorial governments and NGOs) that had different pathways to influence the system	Rogers’ Innovation-Decision Process Model (Rogers 2003) was used to evaluate adoption decisions. No information on a framework guiding scale up reported in the paper
**Gelli et al. (2016 & 2019)** [46, 63]	Diet and malnutrition	Systems approach not described or utilised, systems referred to as ‘monitoring systems’. The intervention combined child-level education and nutrition, alongside household food production. The multi-sectorial intervention sought to link agricultural production with school meals, increasing demand for domestic agriculture and improving school nutrition	Co-ordination and implementation were undertaken by a national secretariat, with programme oversight provided by the Ministry of Local Government and Rural Development. Evaluation involved alternative implementation modalities at scale, acknowledging that implementation relied on interaction between several actors strengthening public institutions and active community engagement. No information on a framework guiding scale up reported in papers
**Hassani et al. (2019)**	Physical activity and diet	Systems approach not described or utilised, system referred to in context of broader childcare system. Had a focus on capacity building among childcare providers, rather than direct intervention, and incorporated multi-segment intervention strategies. Acknowledged the negative influence on implementation as a result of turbulence in the childcare system, and that changes in policies, practices and environments were necessary	Evaluation was guided by the REAIM framework (Glasgow et al. 1999), no information on a scale up framework reported in the paper. Outcomes included early years providers’ capacity to address physical activity. Stakeholder advisory group established to develop the scale up strategy and create a framework for sustainability. Scale up strategy was implemented through province-level not-for-profit partnerships
**Hunt et al. (2020)** [58]	Physical activity, and diet (for the prevention of obesity)	Systems mentioned in the context of the implementation ‘delivery system’, and the study utilised multiple stakeholder organisations. A systems approach was not described, however, there was a focus on capacity building coaches within professional sports clubs, and a train-the-trainer model, to deliver the intervention sustainably. The authors acknowledged that there must be sufficient organisational or system support for effective scale-up	Evaluation was guided by the REAIM framework (Glasgow et al. 1999). The PRACTIS guide (Koorts et al., 2018) (a systems thinking framework to inform implementation and scale up) was retrospectively applied to describe intervention development, evaluation and scale up
**Lonsdale et al. (2016)** [50]	Physical activity	Systems referred to in context of intervention ‘delivery systems’, a systems approach not utilised. The multi-component intervention targeted multiple components in the school environment: quality physical education and school support, classroom movement breaks, physically active homework, active playgrounds, community physical activity links, parent and caregiver engagement	Evaluation was guided by the REAIM framework (Glasgow et al. 1999), no information on a scale up framework reported in the paper
**Rechis et al. (2021)** [61]	Physical activity, tobacco use, diet, UV exposure, inadequate preventive care	Systems referred to in terms of the education system where implementation occurred. Principles of systems thinking, e.g., establishing infrastructure, prioritising target areas, and developing community action plans, was incorporated. Collective impact adopted, which is a collaborative approach to addressing complex social issues	Collective impact framework assessed relational processes, shared goals, and facilitators for successful operations and management of the partnership. No information on a scale up framework reported in the paper
**Wolfenden et al. (2020)** [60]	Physical activity and diet (for the prevention of obesity)	System referred to in terms of an implementation ‘monitoring system’ (a surveillance system). The whole-of-community initiative targeted multiple settings (childcare services, schools, community service organisations, sporting clubs, health services and Aboriginal communities), involving many stakeholders from different sectors. The context of the study was that the intervention sat within and aimed to change broader health system(s). Strong community-academic partnerships were required and acknowledged building capacity, political alignment and significant government funding were essential	Evaluation involved an integrated research-practice approach with co-located researchers and practitioners. Implementation was monitored via a Population Health Intervention Management System. No information on a scale up framework reported in the paper

*NGOs* Non-government organisations, *REAIM* Reach, Effectiveness, Adoption, Implementation and Maintenance framework (Glasgow et al. 1999); *PRACTIS* PRACTical planning for Implementation and Scale up Guide (Koorts et al., 2018)

#### High use of a systems approach to scaling up

Five studies were categorised as demonstrating high use of a systems approach to scaling up [12, 44, 48, 51, 56] (Table 3). Interventions in this group targeted physical activity and diet (*n* = 2), diet (*n* = 1), tobacco use (*n* = 1) and all four behavioural risk factors (*n* = 1). Table 6 provides a case example of a high use systems approach. In these studies, systems thinking informed the intervention design or implementation and scale up approach, or the intervention had a focus on system changes. In these studies, there was an explicit focus on relations between system elements and using system changes to drive impact at scale. Four of the five studies explicitly used systems thinking to inform the intervention design, implementation, and scale up processes, with the goal of systems change (Table 3).

**Table 6:** Case example of a ‘high use’ systems approach to scaling up

**Study:** Tong et al. 2020: The Emergence of a Sustainable Tobacco Treatment Program across the Cancer Care Continuum: A Systems Approach for Implementation at the University of California Davis Comprehensive Cancer Center
**Intervention and scale up context**: A sustainable tobacco treatment programme across a systems framework, targeting smoking reduction among smokers. Scaled up state-wide in California
**Use of a systems approach or term ‘systems’**: This study targeted different systems points, and systems thinking was used to inform implementation. The systems framework was described as a framework that facilitates viewing plans, progress, and priorities. It allowed the identification of research gaps where collaboration was needed to achieve an impact. Systems thinking informed the intervention approach/strategies, and their implementation, as different systems were targeted. The Cancer Care Continuum was adapted as a systems framework, it identified the multiple touchpoints by providers as patients move from primary care to cancer care, with concurrent psychosocial and supportive care that continues survivorship or through to end of life, depending on the patient’s needs. The Systems framework was described as *'a useful framework on which to view plans, progress, and priorities. It helps us identify research gaps, where we must collaborate with others to have an impact, and where more resources may be needed*

##### Conceptualisation and use of ‘systems’ and a systems approach

All five studies in the high use group involved engagement and collaboration with communities and stakeholders in the process and evaluation of scaling up. Four studies explicitly used systems thinking to inform the intervention design, implementation, and scale up processes, with the goal of systems change [12, 48, 51, 56]. Systems thinking was reflected in this process by exploring connections, priorities, and common interests between community groups and organisations [48, 56], identifying systems elements and existing infrastructure that supports scale-up [51], conceptualising systems change in the prevention paradigm and infrastructure [12], and quantifying the connection between systems players and how it contributes to the success of scale-up [44].

##### Methods, theoretical frameworks and/or principles adopted to study scale up processes or outcomes

Three studies in the high use category employed theoretical frameworks to guide the systems thinking practice. One obesity prevention study applied the Analysis Grid for Environments Linked to Obesity (ANGELO) framework [65] to guide the development and implementation of scale-up, defining the key priorities while considering existing capacity within a system [51]. Another study adapted the Cancer Care Continuum as a systems framework to identify the necessary resources and players needed for tobacco treatment integration in each of the cancer care stages (e.g., prevention, diagnosis) [56]. This same study used the Consolidated Framework for Implementation Research (CFIR) [66] to describe and evaluate implementation. The WHO’s Prevention System Building Blocks [67] was applied to one study that targeted all four behavioural risk factors, to identify facilitators and areas for improvement in scale-up of obesity prevention in the domain of workforce, leadership, relationships and networks, resources, and knowledge and data [12]. Three studies in the high use category defined the measures or system level changes resulting from the scale up process [12, 44, 48]. For example, one study used a quantitative systems analysis approach (i.e., social network analysis) to evaluate the diffusion of knowledge of the intervention among stakeholder groups [44], whereas another developed ad-hoc qualitative indicators to capture the presence and/or absence of system-level changes from stakeholders’ perspectives [12].

#### Moderate use of a systems approach to scaling up

Seven studies were categorised as moderate use of a systems approach to scaling up [41–43, 49, 52, 53, 57] (Table 4). Interventions in this group targeted physical activity and diet (*n* = 3), physical activity (*n* = 2), diet (*n* = 1), and tobacco use (*n* = 1). Table 7 provides a case example of a moderate use systems approach. All studies in the group, as described in the papers, recognised the importance of systems thinking in resolving scale-up challenges, however, systems thinking was not embedded from the outset to identify what would be required to achieve system-level changes. System analysis methods were not necessarily used to study or address barriers and facilitators to the scale up process or outcomes. In these studies, there was an implicit focus on systems change, as reflected in the intervention design and evaluation that targeted multiple sectors and components.

**Table 7:** Case example of a ‘moderate use’ systems approach to scaling up

**Study:** Wilcox et al. 2018: Faith, Activity, and Nutrition Randomized Dissemination and Implementation Study: Countywide Adoption, Reach, and Effectiveness
**Intervention and scale up context**: Faith, Activity, and Nutrition (FAN), targets improvements in physical activity and diet among church attendees. Implemented by church committees, at a policy, systems and environmental level. County level implementation across South Carolina, USA, among African Methodist Episcopal churches
**Use of a systems approach or term ‘systems’**: Multicomponent intervention targeting different systems points within faith-based organisations. Acknowledged the role and impact of systems in a systems thinking way, for example, focus was on the church environment, policies and church systems, rather than individual church members. Use of community members/'lay leaders' for implementation

##### Conceptualisation and use of ‘systems’ and a systems approach

For the moderate use group, where systems were involved during scale up, this included via the intervention: (i) *targeting* different system levels (i.e., targeting multilevel organisations) (e.g., [41, 43]), or points of the system (i.e., improving system barriers to access and to affordability) (e.g., [49]); (ii) *involving* organisations that had an influence at different system levels (e.g., schools) (e.g., [53]); or (iii) *including* intervention strategies that had a specific focus on system changes at an organisational (i.e., changing faith based organisations’ engagement in health promotion) (e.g., [57]) or policy/environmental level (i.e., empowering communities) (e.g., [42]). For example, two studies recognised the wide range of determinants on diet and/or physical activity and designed strategies to promote an environment that supports health behaviours (e.g., improve built environment for more opportunities in physical activity) [42, 43]. Further, one study recognised the *interconnectedness* of multiple sectors in smoking cessation and planned for accountability and incentives to support the continued partnership to improve access to cessation services [49].

##### Methods, theoretical frameworks and/or principles adopted to study scale up processes or outcomes

Of the seven included studies, two studies utilised conceptual models of scaling up (i.e., [30, 68]) to guide implementation of interventions targeting physical activity in older adults [52], and in schools [53]. Four of the seven studies described or defined the system level changes resulting from the scale up process [41–43, 52], with a focus on community capacity as an indicator of system level changes. All studies utilised a series of strategies (e.g., educational activities) across multiple sectors (e.g., community, education) to strengthen the community capacity that supports healthy eating and/or physical activity. None of the studies, as reported in the papers, mentioned the measure on the change in community capacity.

#### No use of a systems approach to scaling up

Nine studies were categorised as not using a systems approach to scaling up [45–47, 50, 58–62] (Table 5). Interventions in this group targeted physical activity and diet (*n* = 3), diet (*n* = 2), physical activity (*n* = 2), diet and malnutrition (*n* = 1), and physical activity, tobacco use, diet, UV exposure, and inadequate preventive care (*n* = 1). Table 8 provides a case example. These studies adopted an approach to scaling up that included multiple sectors, settings and intervention components, although, as reported in the papers, the complexity of the system and the relations between the system elements were not explicitly targeted or articulated.

**Table 8:** Case example of a ‘no use’ systems approach to scaling up

**Study:** Hassani et al. 2020, Implementing Appetite to Play at scale in British Columbia: Evaluation of a capacity-building intervention to promote physical activity in the early years
**Intervention and scale up context:** Appetite to Play, a capacity building initiative for early years providers to implement policies, practices and environments that support physical activity and healthy eating. Scaled up at the province level, in British Columbia (BC), Canada, via Child Health BC and multiple not-for-profit organisations. A train the trainer model was used for dissemination
**Use of systems approach or term ‘systems’**: The broader context of challenges in the childcare system were acknowledged, but a systems approach was not described or utilised. There was a focus on capacity building among childcare providers within the childcare system, and a provincial stakeholder advisory group was set up to advise the partnership group on the development of both the resource, scale-up strategy, implementation (course correction), and to create a framework for sustainability

##### Conceptualisation and use of ‘systems’ and a systems approach

Where systems were involved during scale up in the ‘no use’ group, this included via the intervention: (i) *targeting* different system levels (i.e., targeting multilevel organisations, e.g., [62]) or settings within the system (e.g., [60]); (ii) *involving* organisations that had an influence at different system levels (e.g., representing food retailers, Non-farmers, advertisers) (e.g., [45, 46, 59]); or (iii) *including* intervention strategies that had a specific focus on system changes at an organisational level (i.e., changing childcare provider capacity to engage in health promotion or increasing football club coaches capacity to deliver health promotion within their existing organisations) (e.g., [47, 58]). All studies in this group involved multiple strategies in multiple sectors and may have involved targeting different systems; however, the strategies and sectors were not considered in a relational way or tended to focus on one of few points of within the system. For example, in one study, only school meals service and distribution were focussed on, even though the intervention was designed as a multi-sectoral strategy to increase food production, household income, and food security [46]. Several multicomponent interventions did not describe or explicitly target systems change (e.g., [50]), but acknowledged the role and influence of system factors (e.g., [47]). Whilst some studies acknowledged that there must be sufficient organisational or system support for effective scale up, where the term ‘system(s)’ was mentioned, this was referred in context of: ‘evaluation systems’ (e.g., [62]), ‘monitoring systems’ (e.g., [46, 60]), implementation ‘delivery system’ (e.g., [50, 58]), or in the broader general context of the social/childcare/health system that the intervention took place (e.g., [45, 59]). Where ‘systems’ were not explicitly mentioned, factors relevant to systems change could still be utilised (i.e., establishing community infrastructure and developing community action plans to support capacity at scale) (e.g., [61]) (Table 5).

##### Methods, theoretical frameworks and/or principles adopted to study scale up processes or outcomes

None of the nine studies included in the ‘no use’ group described using a scale up framework. One study retrospectively applied the PRACTIS guide [69], which is a framework that incorporates a systems thinking perspective on effective implementation and scale up, to describe the scale up of an intervention targeting professional football clubs [58]. However, the authors acknowledge that the PRACTIS guide was not available to guide intervention scale up from the initial stages. Concepts within the social system, relevant to Rogers’ Diffusion of Innovations [70], informed the evaluation of two studies, to establish scalability of an intervention [59] and actual scale up outcomes [45]. Five of the nine studies described system level changes resulting from scale up, mainly on outcomes as a result of multiple strategies implemented in interventions [47, 50, 61, 62] or as a result of strong policy alignment [60].

#### Quality appraisal findings

Quality assessment findings are presented in Additional file 3. In general, there were a lack of details in the reporting of the sampling strategy and whether the study sample is representative of the target population, and the risk of nonresponse bias for quantitative survey was rarely considered. For mixed methods studies, it was often unclear how divergences and inconsistencies between quantitative and qualitative results were addressed.

## Discussion

Systems approaches and complexity science have potential for enhancing both the development and the scaling up of health interventions [20, 21, 71], but there is a paucity of knowledge of whether and how systems approaches have been adopted when scaling up in public health [14]. In this paper, we systematically explore the use of systems approaches to scaling up prevention of four behavioural risk factors for NCDs; physical inactivity, tobacco use, alcohol consumption and unhealthy diet. Of the studies included in this review, interventions targeted a mix of behavioural risk factors across different ages and settings, however, almost all studies were conducted in high income countries. Despite the fact that the majority of interventions in this review were described as having been designed for scale, only four (19%) of the 21 interventions explicitly used systems thinking to inform the intervention design, implementation, and scale up processes, with the goal of systems change. Whilst studies often included multiple sectors and intervention components; recognition of the complexity of the system and the relations between the system elements were not explicitly targeted or articulated.

Systems approaches are not imperative for scale up, and there is a lack of robust evidence that a systems approach leads to better outcomes for sustainability and impact at scale. However, this review illustrates that current conceptualisations of what constitutes designing an intervention for scale does not necessarily include a consideration of the impact of systems or principles of systems thinking. This is despite the fact that successful scale up includes when an evidence-based intervention becomes embedded in a system(s) to achieve long-term, sustainable health impact [10]. We also identified that interventions described as designed for scale were reliant on strong research-practice partnerships. Facilitators to scale up included committed stakeholder engagement, capacity building in the local workforce, and flexibility in delivery and implementation. Conversely, barriers included competing interests and priorities, and insufficient time and resources. These barriers and facilitators are consistent with previous scale up research [72, 73]. Studies in this review also reported challenges relating to measuring system changes, including the time required to detect long-term changes [51], and challenges with appropriate measures for population reach and engagement [48], which is consistent with research that explored some of the tensions of scaling up in physical activity [13].

More than two thirds of interventions included were described as designed for scale, whereas less than one third of interventions followed a traditional translation pathway of small-scale controlled efficacy testing, to effectiveness and implementation at scale. This finding suggests that there may have been a move away from the traditional translation pipeline that begins with controlled research trials, to one that considers effective real-world translation, as has been recommended for more than two decades [74]. It also suggests that there is greater consideration of translation and population impact early in the research process, which has also been recommended for over a decade [75]. Nonetheless, despite recommendations for the use of complex systems models in public health [20], recognition of the impact of system factors varied greatly across studies. Of those studies that did adopt a systems approach to scale up (the ‘high use’ group), most of the interventions targeted all four behavioural risk factors for NCDs we included in this review. In this high use group, there was an explicit focus on relations between system elements and using system changes to drive impact at scale. Of the studies that did not adopt a systems approach (the ‘no use’ group), most of the interventions targeted physical activity and diet.

The varied appreciation, adoption and implementation of systems approaches for the four NCD behavioural risk factors we included in this review may reflect the overall lack of scale up approaches that adopted systems perspectives. For example, in physical activity promotion research, systems approaches have largely been underutilised and systems concepts need to be engaged more robustly in physical activity interventions [24]. In physical activity *scale up* research in particular, systems approaches are perceived as important, but may not be seen as feasible to achieve in practice [10]. In obesity prevention research, which can include interventions targeting physical activity and diet, there is a lack of evidence for whole of systems approaches [34]. Complex systems perspectives have been applied to study alcohol reduction and associated harms, however their application has remained predominantly at the individual or local level [19]. Consistent with public health promotion more broadly [20], alcohol reduction interventions have often been reductionist with a focus on easily modifiable risk factors and high risk groups [76], despite advocacy for a focus on the real-world systems that alcohol consumption and harms are created and shaped by [19]. For smoking cessation, the benefits of approaches that systemically integrate into or incorporate clinical settings have long been recognised, however, their adoption has been inconsistent and implementation slow [77]. For several decades, tobacco control has become increasingly complex and thus the use of systems thinking has long been encouraged [78]. However, the effectiveness of system change interventions on tobacco cessation rates and system level outcomes at scale, remains less clear [79].

Whilst our review showed that socio-ecological models have been an integral part of designing, implementing, and evaluating physical activity interventions, the interconnectedness among these elements in scale up was rarely considered. Implementation of a suite of activities across multiple settings or levels need not mean a systems approach has been adopted [80]. Our findings support this, as studies in both the high and no use systems approach groups included interventions targeting multiple settings and levels. Yet, only studies in the high use group (e.g., [48]) explicitly applied systems thinking in practice and embedded systems approaches from the outset.

Consistent with the definition of a systems approach to scale up that we used in this paper [14], scale up exists on a continuum and scale up need not adopt a systems approach. As is shown in this review, scale up approaches can include linear, intervention-orientated expansive approaches that prioritise the spread of interventions *into* existing systems, through to approaches that sit within a complex systems paradigm that begin by considering the characteristics of the target system(s) that scaling occurs within [14]. Scaling health interventions in a traditional, linear way through efficacy to effectiveness and scale up trials is well documented (e.g., [73]). Our review draws attention to the fact that there is a lack of published evidence for ways to operationalise systems approaches when scaling interventions, including how to select and apply relevant theories and frameworks. Of the 21 interventions we included, only three employed a theoretical framework to guide systems thinking practice. Of the studies that did, to some extent, adopt a systems approach to scale up (i.e., the high and moderate use groups), system-level change and the scale up processes and outcomes that drive the change at scale were not well defined, and often the reporting was brief and lacked theoretical explanation. When a systems approach was applied and concisely reported, it provided a more comprehensive view of a problem and can help identify potential barriers and opportunities for scaling up interventions (e.g., [56]), however, our quality appraisal highlighted that most studies lacked information on key parameters.

To progress the field and better equip researchers, practitioners and policymakers to invest in efforts for scaling up, it has been recommended that NCD prevention adopts new paradigms and perspectives that incorporate systems thinking [18, 20, 81]. This includes using frameworks that incorporate a systems thinking perspective on how to achieve effective outcomes at scale [13] and adopt new ways of accounting for the complex systems in which interventions are implemented [82]. Complexity and systems theory can be used to understand and approach population health scale-up by considering the system as a whole prior to intervention design, development and implementation. For example, Scaling Readiness assessments involve both quantitative and qualitative data collection, and can be used to ascertain the characteristics of the system that influence prospective interventions prior to investment [83]. The Intervention Scalability Assessment Tool (ISAT) [84] has also been used to assess prospective scalability of interventions into existing systems, through a participatory process with stakeholders. For frameworks that guide scale up planning, the PRACTIS guide [69] adopts a systems thinking perspective to scaling up that can inform both scale up planning [85] and evaluation [58]. Specifically, PRACTIS workshops (i.e., [86]) involve a participatory process and co-design process with stakeholders, which allows for the systematic identification and documentation of data that are influential for intervention uptake, political support and community sustainability across multiple levels of the system. For data collection that can account for the complex systems in which interventions are implemented, resources such as the Consolidated Framework for Implementation Research (CFIR) [66] outline key factors influencing implementation of interventions, including ways of collecting data to account for these multilevel factors at scale. A key component of the Designing for Dissemination and Sustainability (D4DS) logic model [85], is designing a research product with the end in mind. This includes, understanding the characteristics of systems by using methods (e.g., system dynamics modelling [87]) to anticipate and plan for adaptation of interventions in response to changes in context over time [85]. Use of these frameworks and tools is recommended for improving operationalisation of systems approaches in the context of public health scale up.

Building the evidence base in systems approaches to scaling up, for physical activity and other NCD behavioural risk factors, has the potential to improve how we communicate and operationalise systems approaches when scaling for widespread and sustainable impact, in both research and in practice.

## Strengths and limitations

A key strength of this review is that we utilised a narrative synthesis approach which enabled us to account for different scale up approaches and the varied terminology used to describe systems and systems approaches. Due the complicated and understudied nature of the topic we address in this paper, and thus the extended time required to extract, analyse, and reach consensus on the use of a systems approach to scaling up; we acknowledge the gap between data extraction and publishing. However, by combining a narrative synthesis approach with systematic screening, independent data categorisation, and quality assessment of methodological rigour, the robustness of our review is enhanced. The data were extracted by two independent researchers, and the analytical framework was developed, and data synthesised by three independent researchers. Given the lack of a universally agreed-upon definition of a 'systems approach' and the broad use of systems language, our methodology enabled us to capture implicit and explicit systems methods and approaches. Our analytical framework also allowed us to identify whether scaling up adhered to a systems approach, even if the term 'systems approach' was not explicitly used in the study.

Our systematic search process meant that we were able to identify relevant articles, however, as our data synthesis was reliant on information contained within the published papers, it is unknown to what extent systems or systems approaches were considered in the scale up process by decision-makers but were simply not reported in the published articles. We undertook reference list searches for studies where information was incomplete or unclear, however, this was only conducted for the scale up strategy and framework used, and not for all aspects of scale up. There is thus the potential that additional information related to scale up may have been published elsewhere. In addition, we also only report on the country of scale up directly relevant to the papers included, whereas interventions may have been expanded to other countries elsewhere. For example, for one intervention we acknowledge that adaptations had occurred for implementation in other countries (e.g., [58]), which we do not report on in our results. In this review, we focused on the four major behavioural risk factors for NCDs [2]. We acknowledge that the WHO physical activity guidelines also include recommendations related to sedentary behaviour [88]. Sedentary behaviour is considered a distinct health risk factor to physical inactivity [89, 90], and thus future reviews may wish to broaden the scope of included studies to align with global guidelines. To our knowledge, this review is the first to categorise the use of systems approaches in scale up, however, future research is needed to quantify different levels. Finally, non-English publications were excluded from this review.

## Conclusion

Systems approaches allow for consideration of complexity at scale, facilitating different ways of planning for and interpreting challenges that are associated with scale up. Systems approaches also align with some of the key facilitators to successful scale up. By acknowledging the interconnectedness among various components of a system and ensuring their efficient and effective collaboration; systems approaches can potentially lead to more successful and impactful scale up outcomes. This review showed that the use of systems approaches when scaling up interventions targeting key behavioural risk factors for NCDs (physical inactivity, tobacco use, alcohol consumption and diet) is still in its infancy and there is a need for high quality studies. In particular for population level physical activity promotion, this presents a huge gap in knowledge. For decision-makers wishing to adopt or support a systems approach to intervention implementation at scale, greater guidance is needed on what is required to achieve this, and how to communicate and operationalise systems approaches in both research and practice.

### Supplementary Information

**Additional file 1.** PRISMA Checklist.

**Additional file 2.** Online database search strategy.

**Additional file 3.** The Mixed Methods Appraisal Tool findings.

## Data Availability

The datasets used and/or analyzed during the current study are available from the corresponding author on reasonable request.

## Abbreviations

NCD: Non-communicable disease
WHO: World Health Organization

## References

[1] Global Burden of Disease Collaborative Network, Global Burden of Disease Study 2019 (GBD 2019) Results (2020, Institute for Health Metrics and Evaluation – IHME) https://vizhub.healthdata.org/gbd-results/. 2019.

[2] World Health Organization (2013). Global action plan for the prevention and control of noncommunicable diseases 2013–2030.

[3] World Health Organization (2018). Global Action Plan on Physical Activity 2018–2030: more active people for a healthier world.

[4] World Health Organization (2022). Global Alcohol Action Plan 2022–2030.

[5] World Health Organization (2022). Global status report on physical activity 2022.

[6] https://www.who.int/news-room/fact-sheets/detail/alcohol. Cited 2023 31st March.

[7] https://www.who.int/news-room/facts-in-pictures/detail/6-facts-on-obesity. Cited 2023 31st March.

[8] Santos AC (2023). The cost of inaction on physical inactivity to public health-care systems: a population-attributable fraction analysis. Lancet Glob Health.

[9] on, W.H.O.I.H.-l.C. and NCDs. Think piece: why is 2018 a strategically important year for NCDs? Geneva: World Health Organization; 2018.

[10] Reis RS (2016). Scaling up physical activity interventions worldwide: stepping up to larger and smarter approaches to get people moving. Lancet.

[11] Murphy J (2023). Advocating for implementation of the global action plan on physical activity: challenges and support requirements. J Phys Act Health.

[12] Matheson A (2020). Strengthening prevention in communities through systems change: lessons from the evaluation of Healthy Families NZ. Health Promot Int.

[13] Koorts H (2022). Tensions and paradoxes of scaling up: a critical reflection on physical activity promotion. Int J Environ Res Public Health.

[14] Koorts H, Rutter H (2021). A systems approach to scale-up for population health improvement. Health Res Pol Syst.

[15] Lane C (2021). How effective are physical activity interventions when they are scaled-up: a systematic review. Int J Behav Nutr Phys Act.

[16] Koorts H (2021). Mechanisms of scaling up: combining a realist perspective and systems analysis to understand successfully scaled interventions. Int J Behav Nutr Phys Act.

[17] Rutter H (2019). Systems approaches to global and national physical activity plans. Bull World Health Organ.

[18] Allender S (2019). Translating systems thinking into practice for community action on childhood obesity. Obes Rev.

[19] McGill E (2021). Applying a complex systems perspective to alcohol consumption and the prevention of alcohol-related harms in the 21st century: a scoping review. Addiction.

[20] Rutter H (2017). The need for a complex systems model of evidence for public health. Lancet.

[21] Paina L, Peters DH (2012). Understanding pathways for scaling up health services through the lens of complex adaptive systems. Health Policy Plan.

[22] Komashie A (2021). Systems approach to health service design, delivery and improvement: a systematic review and meta-analysis. BMJ Open.

[23] Fiore MC, Keller PA, Curry SJ (2007). Health system changes to facilitate the delivery of tobacco-dependence treatment. Am J Prev Med.

[24] Nau T (2022). A scoping review of systems approaches for increasing physical activity in populations. Health Res Pol Syst.

[25] Jebb SA, et al. Systems-based approaches in public health: Where next?. Canadian Academy of Health Sciences & The Academy of Medical Sciences; 2021.

[26] Carey G (2015). Systems science and systems thinking for public health: a systematic review of the field. BMJ Open.

[27] Bolton KA (2022). Generating change through collective impact and systems science for childhood obesity prevention: The GenR8 change case study. Plos One.

[28] Page MJ (2021). The PRISMA 2020 statement: an updated guideline for reporting systematic reviews. BMJ.

[29] Peters DH (2014). The application of systems thinking in health: why use systems thinking?. Health Res Pol Syst.

[30] Simmons R, Shiffman J (2007). Scaling up health service innovations: a framework for action, in scaling up health service delivery.

[31] Indig D (2017). Pathways for scaling up public health interventions. BMC Public Health.

[32] Rabin BA (2008). A glossary for dissemination and implementation research in health. J Public Health Manag Pract.

[33] Rychetnik L (2002). Criteria for evaluating evidence on public health interventions. J Epidemiol Community Health.

[34] Bagnall AM (2019). Whole systems approaches to obesity and other complex public health challenges: a systematic review. BMC Public Health.

[35] Palmer M (2018). The effectiveness of smoking cessation, physical activity/diet and alcohol reduction interventions delivered by mobile phones for the prevention of non-communicable diseases: a systematic review of randomised controlled trials. Plos One.

[36] Rehm J (2019). Alcohol use and dementia: a systematic scoping review. Alzheimer's Res Ther.

[37] McKenzie JE, et al. Defining the criteria for including studies and how they will be grouped for the synthesis. In: Higgins JPT, et al., editors. Cochrane Handbook for Systematic Reviews of Interventions. England: Cochrane; 2022.

[38] Riva JJ (2012). What is your research question? An introduction to the PICOT format for clinicians. J Can Chiropr Assoc.

[39] Hong QN (2018). The Mixed Methods Appraisal Tool (MMAT) version 2018 for information professionals and researchers. Educ Inf.

[40] Popay J, et al. Guidance on the conduct of narrative synthesis in systematic reviews: A product from the ESRC Methods Programme. 2006. https://citeseerx.ist.psu.edu/document?repid=rep1&type=pdf&doi=ed8b23836338f6fdea0cc55e161b0fc5805f9e27.

[41] Berman M (2018). Evaluation of the healthy lifestyles initiative for improving community capacity for childhood obesity prevention. Prev Chronic Dis.

[42] Betancourt K (2017). Empowering one community at a time for policy, system and environmental changes to impact obesity. Ethn Dis.

[43] Bolton KA (2017). The outcomes of health-promoting communities: being active eating well initiative-a community-based obesity prevention intervention in Victoria Australia. Int J Obes (Lond).

[44] Conte KP (2017). Dynamics behind the scale up of evidence-based obesity prevention: protocol for a multi-site case study of an electronic implementation monitoring system in health promotion practice. Implement Sci.

[45] Fernandez MA (2016). Factors influencing the adoption of a healthy eating campaign by federal cross-sector partners: a qualitative study. BMC Public Health.

[46] Gelli A (2019). A school meals program implemented at scale in Ghana increases height-for-age during midchildhood in girls and in children from poor households: a cluster randomized trial. J Nutr.

[47] Hassani K (2020). Implementing appetite to play at scale in British Columbia: evaluation of a capacity-building intervention to promote physical activity in the early years. Int J Environ Res Public Health.

[48] Joyce A (2018). The 'Practice Entrepreneur' - An Australian case study of a systems thinking inspired health promotion initiative. Health Promot Int.

[49] Livingston CJ (2020). Reducing tobacco use in Oregon through multisector collaboration: aligning medicaid and public health programs. Prev Chronic Dis.

[50] Lonsdale C (2016). Scaling-up an efficacious school-based physical activity intervention: study protocol for the 'Internet-based Professional Learning to help teachers support Activity in Youth' (iPLAY) cluster randomized controlled trial and scale-up implementation evaluation. BMC Public Health.

[51] Malakellis M (2017). School-based systems change for obesity prevention in adolescents: outcomes of the Australian Capital territory 'It's Your Move!'. Aust N Z J Public Health.

[52] McKay HA (2021). Status quo or drop-off: do older adults maintain benefits from choose to move-a scaled-up physical activity program-12 months after withdrawing the intervention?. J Phys Act Health.

[53] Nettlefold L (2021). Scaling up action schools! BC: how does voltage drop at scale affect student level outcomes? A cluster randomized controlled trial. Int J Environ Res Public Health.

[54] Sacher PM (2019). Addressing childhood obesity in low-income, ethnically diverse families: outcomes and peer effects of MEND 7–13 when delivered at scale in US communities. Int J Obes.

[55] Sutherland R (2020). Scale-up of the Physical Activity 4 Everyone (PA4E1) intervention in secondary schools: 24-month implementation and cost outcomes from a cluster randomised controlled trial. Int J Behav Nutr Phys Act.

[56] Tong EK (2020). The emergence of a sustainable tobacco treatment program across the cancer care continuum: a systems approach for implementation at the university of California Davis comprehensive cancer center. Int J Environ Res Public Health.

[57] Wilcox S (2018). Faith, activity, and nutrition randomized dissemination and implementation study: countywide adoption, reach, and effectiveness. Am J Prev Med.

[58] Hunt K (2020). Scale-up and scale-out of a gender-sensitized weight management and healthy living program delivered to overweight men via professional sports clubs: the Wider Implementation of Football Fans in Training (FFIT). Int J Environ Res Public Health.

[59] Blake MR (2021). The ‘Eat Well @ IGA’ healthy supermarket randomised controlled trial: process evaluation. Int J Behav Nutr Phys Act.

[60] Wolfenden L (2020). From demonstration project to changes in health systems for child obesity prevention: the legacy of 'Good for Kids, Good for Life'. Aust N Z J Public Health.

[61] Rechis R (2021). Be Well Communities™: mobilizing communities to promote wellness and stop cancer before it starts. Cancer Causes Control.

[62] Davis SM, Cruz TH, Kozoll RL (2017). Research to practice: implementing physical activity recommendations. Am J Prev Med.

[63] Gelli A (2016). Evaluation of alternative school feeding models on nutrition, education, agriculture and other social outcomes in Ghana: rationale, randomised design and baseline data. Trials.

[64] The World Bank Group, High income data. The World Bank Group; 2019.

[65] Swinburn B, Egger G, Raza F (1999). Dissecting obesogenic environments: the development and application of a framework for identifying and prioritizing environmental interventions for obesity. Prev Med.

[66] Damschroder LJ (2009). Fostering implementation of health services research findings into practice: a consolidated framework for advancing implementation science. Implement Sci.

[67] World Health Organization (2007). Everybody's business - strengthening health systems to improve health outcomes: WHO's framework for action.

[68] Yamey G (2011). Scaling up global health interventions: a proposed framework for success. Plos Med.

[69] Koorts H (2018). Implementation and scale up of population physical activity interventions for clinical and community settings: the PRACTIS guide. Int J Behav Nutr Phys Act.

[70] Rogers E (2003). Diffusion of Innovations.

[71] Greenhalgh T, Papoutsi C (2019). Spreading and scaling up innovation and improvement. BMJ.

[72] Mangham LJ, Hanson K (2010). Scaling up in international health: what are the key issues?. Health Policy Plan.

[73] World Health Organization (2009). Practical guidance for scaling up health service innovations.

[74] Glasgow R, Lichtenstein E, Marcus A (2003). Why don't we see more translation of health promotion research to practice? Rethinking the efficacy-to-effectiveness transition. Am J Public Health.

[75] World Health Organization (2011). Beginning with the end in mind: planning pilot projects and other programmatic research for successful scaling up.

[76] Atkinson J-A (2018). Harnessing advances in computer simulation to inform policy and planning to reduce alcohol-related harms. Int J Public Health.

[77] Pipe AL, Evans W, Papadakis S (2022). Smoking cessation: health system challenges and opportunities. Tob Control.

[78] National Cancer Institute (2007). Greater than the sum: systems thinking in Tobacco control.

[79] Thomas D (2017). System change interventions for smoking cessation. Cochrane Database Syst Rev.

[80] Hawe P, Shiell A, Riley T (2009). Theorising interventions as events in systems. Am J Community Psychol.

[81] Braithwaite J (2018). When complexity science meets implementation science: a theoretical and empirical analysis of systems change. BMC Med.

[82] Jago R (2023). Rethinking children’s physical activity interventions at school: a new context-specific approach. Front Public Health.

[83] Sartas M (2020). Scaling readiness: science and practice of an approach to enhance impact of research for development. Agric Syst.

[84] Lee K (2020). The intervention scalability assessment tool: a pilot study assessing five interventions for scalability. Public Health Res Pract.

[85] Kwan BM, Luke DA, Adsul P, Koorts H, Morrato EH, Glasgow RE, Brownson RC, Colditz GA, Proctor EK (2023). Designing for Dissemination and Sustainability: Principles, Methods, and Frameworks for Ensuring Fit to Context. Dissemination and Implementation Research in Health.

[86] Hesketh KD (2022). Protocol for the Let’s Grow randomised controlled trial: examining efficacy, cost-effectiveness and scalability of a m-Health intervention for movement behaviours in toddlers. BMJ Open.

[87] Sterman JD (2000). Business Dynamics: Systems Thinking and Modeling for a Complex World.

[88] World Health Organization (2020). WHO guidelines on physical activity and sedentary behaviour.

[89] Biddle SJ (2016). Too much sitting and all-cause mortality: is there a causal link?. BMC Public Health.

[90] Owen N (2020). Sedentary behavior and public health: integrating the evidence and identifying potential solutions. Annu Rev Public Health.

