# Supplementary material for: Systems approaches to scaling up: a systematic review and narrative synthesis of evidence for physical activity and other behavioural non-communicable disease risk factors
Source: Int J Behav Nutr Phys Act. 2024 Mar 21;21:32. doi: 10.1186/s12966-024-01579-6 (PMC10958859; doi:10.1186/s12966-024-01579-6)
Supplement: Supplementary file 2 — Additional file 2. Online database search strategy. [file 12966_2024_1579_MOESM2_ESM.docx]

**Additional File 3.** Online databases and search strings

*Online database search*

via EBSCO host, CINAHL complete, Medline complete, Global Health, PsychINFO and Sport Discuss and via EMBASE. The searches were conducted in October, 2021 returned 70,473 hits using the following search string; implement* OR disseminat* OR diffusion OR “scaleup” OR “scaled up” OR “roll* out” OR expand* OR “scaling up” OR upscaling OR “up scaling” OR “at scale” OR scaling OR scalable OR “scaling out” OR scalability OR translat* OR institutionali?ation OR adopt* OR uptake OR utili?ation AND "whole-systems approach" OR holisitc OR "cross sector" OR " systems based" OR "systems approach" OR "multi-strategy approach*" OR " systems analysis" OR "systems theory" OR "agent based" OR "system dynamic*" OR model* OR "system* model" OR "system* map*" OR network* OR "system* science" OR "system* methodolog*" OR "systems thinking" OR "systems dynamic" OR "complex system*"  AND approach* OR strateg* OR initiative* OR scheme* OR program* OR intervention* OR prevent*  AND ("physical * activ*" OR "physical* inactiv*" OR exercis* OR sport* OR fitness OR recreation* OR "leisure active*" OR "active play*" OR walk* OR cycl* OR bicycl* OR biking OR bike OR "active transport" OR "active travel" OR "active commu*" OR "active living" OR "sedentary lifestyle" OR sedentar* OR "sedentary behavio*" OR sitting) ( obes* OR overweight OR "body weight" OR diet* OR nutrition* OR overeat* OR "health* weight" OR "health* eat*" OR "health* choice*" OR adiposity OR "weight gain*" OR "weight change*" OR "weight retention*" OR "weight loss*" OR "bmi gain" OR "bmi loss" OR "bmi change" OR "body mass index gain" OR "body mass index change" OR "body mass index loss") ( tobacco OR smok* OR cigarette* OR nicotine OR "tobacco use" OR "tobacco use cessation" OR "smoking cessation" OR "tobacco depend*" OR "tobacco addict*") ( "alcohol abuse" OR "alcohol related disorders" OR "alcohol induced disorder*" OR "alcohol* intoxicat*" OR "alcohol withdraw*" OR "alcohol drink*" OR abstinen* OR abstain* OR " alcohol addict*" OR dependen* OR detox* OR excessiv* OR "alcohol related harm*" OR "heavy drink*" OR "high risk drink*" OR "alcohol misus*" OR "drinking behav*" OR "binge drinking" OR "alcohol drinking patterns" OR "alcoholic intoxication" OR "alcohol consumption")

*Grey literature search*

An advanced Google search using the following search strings. The first 10 pages were reviewed: search 1 (19 results): All of these words: physical activity OR "whole-systems approach" "scale up" OR "roll out" Any of these words: (scale OR scaling OR roll-out OR adopt OR translation) (intervention OR program OR initiative OR population OR community OR government); search 2 (10 results) : All of these words: obesity OR overweight OR "healthy eating" OR "whole-systems approach" "scale up" OR "roll out" Any of these words: (scale OR scaling OR roll-out OR adopt OR translation) (intervention OR program OR initiative OR population OR community OR government); search 3 (6 results): All of these words: tobacco OR smoke OR cigarette OR nicotine OR "tobacco use" scale-up intervention Any of these words: whole-systems approach OR roll-out OR adopt OR translation OR program; search 4 (3 results): All of these words: alcohol-related harm OR "drinking behaviour" OR "alcohol drinking patterns" scale-up intervention Any of these words: whole-systems approach OR roll-out OR adopt OR translation. These searches combined returned over 800,000 hits on the 3rd of November 2021.
