# Supplementary material for: Systems approaches to scaling up: a systematic review and narrative synthesis of evidence for physical activity and other behavioural non-communicable disease risk factors
Source: Int J Behav Nutr Phys Act. 2024 Mar 21;21:32. doi: 10.1186/s12966-024-01579-6 (PMC10958859; doi:10.1186/s12966-024-01579-6)
Supplement: Supplementary file 3 — Additional file 3. The Mixed Methods Appraisal Tool findings. [file 12966_2024_1579_MOESM3_ESM.docx]

**Additional File 4.** Results of quality appraisal of included studies using The Mixed Methods Appraisal Tool (MMAT)

| Author (Year) | Screening questions (for all) | | | 1. Qualitative | | | | | | 2. Quantitative randomized  controlled trials | | | | | | 3. Quantitative non-randomized | | | | | | 4. Quantitative descriptive | | | | | | 5. Mixed methods | | | | | |
| --- | --- | --- | --- | --- | --- | --- | --- | --- | --- | --- | --- | --- | --- | --- | --- | --- | --- | --- | --- | --- | --- | --- | --- | --- | --- | --- | --- | --- | --- | --- | --- | --- | --- |
|  | S1 | S2 | 1.1 | | 1.2 | 1.3 | 1.4 | 1.5 | 2.1 | | 2.2 | 2.3 | 2.4 | 2.5 | 3.1 | | 3.2 | 3.3 | 3.4 | 3.5 | 4.1 | | 4.2 | 4.3 | 4.4 | 4.5 | 5.1 | | 5.2 | 5.3 | 5.4 | 5.5 |  |
| Berman et al. (2018) | Y | Y |  | |  |  |  |  |  | |  |  |  |  |  | |  |  |  |  |  | |  |  |  |  | Y | | Y | Y | Y | Y |  |
| Betancourt et al. (2017) | Y | Y |  | |  |  |  |  |  | |  |  |  |  |  | |  |  |  |  |  | |  |  |  |  | Y | | Y | Y | ? | N |  |
| Blake et al. (2021) | Y | Y |  | |  |  |  |  |  | |  |  |  |  |  | |  |  |  |  |  | |  |  |  |  | Y | | Y | Y | ? | Y |  |
| Bolton et al. (2017) | Y | Y |  | |  |  |  |  |  | |  |  |  |  |  | |  |  |  |  | Y | | N | Y | Y | Y |  | |  |  |  |  |  |
| Conte et al. (2017) | Y | ? (protocol) |  | |  |  |  |  |  | |  |  |  |  |  | |  |  |  |  |  | |  |  |  |  |  | |  |  |  |  |  |
| Davis et al. (2017) | Y | Y |  | |  |  |  |  |  | |  |  |  |  |  | |  |  |  |  |  | |  |  |  |  | Y | | Y | ? | ? | ? |  |
| Fernandez et al. (2016) | Y | Y | Y | | Y | Y | Y | Y |  | |  |  |  |  |  | |  |  |  |  |  | |  |  |  |  |  | |  |  |  |  |  |
| Gelli et al. (2016) | Y | ? (protocol) |  | |  |  |  |  |  | |  |  |  |  |  | |  |  |  |  |  | |  |  |  |  |  | |  |  |  |  |  |
| Gelli et al. (2019) | Y | Y |  | |  |  |  |  | Y | | Y | ? | N | Y |  | |  |  |  |  |  | |  |  |  |  |  | |  |  |  |  |  |
| Hassani et al. (2020) | Y | Y |  | |  |  |  |  |  | |  |  |  |  |  | |  |  |  |  |  | |  |  |  |  | Y | | Y | Y | ? | Y |  |
| Hunt et al. (2020) | Y | Y |  | |  |  |  |  |  | |  |  |  |  |  | |  |  |  |  |  | |  |  |  |  | Y | | Y | Y | ? | Y |  |
| Joyce et al. (2018) | Y | Y | Y | | Y | Y | Y | Y |  | |  |  |  |  |  | |  |  |  |  |  | |  |  |  |  |  | |  |  |  |  |  |
| Livingston et al. (2020) | Y | Y |  | |  |  |  |  |  | |  |  |  |  |  | |  |  |  |  | ? | | Y | Y | ? | ? |  | |  |  |  |  |  |
| Lonsdale et al. (2016) | Y | ? (protocol) |  | |  |  |  |  |  | |  |  |  |  |  | |  |  |  |  |  | |  |  |  |  |  | |  |  |  |  |  |
| Malakellis et al. (2017) | Y | Y |  | |  |  |  |  |  | |  |  |  |  | Y | | Y | N | Y | ? |  | |  |  |  |  |  | |  |  |  |  |  |
| Matheson et al. (2019) | Y | Y |  | |  |  |  |  |  | |  |  |  |  |  | |  |  |  |  |  | |  |  |  |  | Y | | Y | Y | ? | Y |  |
| McKay et al. (2021) | Y | Y |  | |  |  |  |  |  | |  |  |  |  |  | |  |  |  |  | Y | | Y | Y | N | Y |  | |  |  |  |  |  |
| Nettlefold et al. (2021) | Y | Y |  | |  |  |  |  | Y | | Y | Y | N | Y |  | |  |  |  |  |  | |  |  |  |  |  | |  |  |  |  |  |
| Rechis et al. (2021) | Y | Y |  | |  |  |  |  |  | |  |  |  |  |  | |  |  |  |  | ? | | ? | Y | ? | Y |  | |  |  |  |  |  |
| Tong et al. (2020) | Y | Y | Y | | ? | Y | Y | Y |  | |  |  |  |  |  | |  |  |  |  |  | |  |  |  |  |  | |  |  |  |  |  |
| Wilcox et al. (2018) | Y | Y |  | |  |  |  |  | Y | | Y | Y | ? | ? |  | |  |  |  |  |  | |  |  |  |  |  | |  |  |  |  |  |
| Wolfenden et al. (2020) | Y | ? (editorial) |  | |  |  |  |  |  | |  |  |  |  |  | |  |  |  |  |  | |  |  |  |  |  | |  |  |  |  |  |

Note. Y = Yes, N = No, ? = Can’t tell.

MMAT sourced from: Hong, Q.N., Fàbregues, S., Bartlett, G., Boardman, F., Cargo, M., Dagenais, P., Gagnon, M.P., Griffiths, F., Nicolau, B., O’Cathain, A. and Rousseau, M.C., 2018. The Mixed Methods Appraisal Tool (MMAT) version 2018 for information professionals and researchers. Education for information, 34(4), pp.285-291.

**Methodological quality criteria**

S1 Are there clear research questions?

S2 Do the collected data allow to address the research questions?

1. Qualitative

1.1 Is the qualitative approach appropriate to answer the research question?

1.2 Are the qualitative data collection methods adequate to address the research question?

1.3 Are the findings adequately derived from the data?

1.4 Is the interpretation of results sufficiently substantiated by data?

1.5 Is there coherence between qualitative data sources, collection, analysis and interpretation?

2. Quantitative randomized controlled trials

2.1 Is randomization appropriately performed?

2.2 Are the groups comparable at baseline?

2.3 Are there complete outcome data?

2.4 Are outcome assessors blinded to the intervention provided?

2.5 Did the participants adhere to the assigned intervention?

3. Quantitative non-randomized

3.1 Are the participants representative of the target population?

3.2 Are measurements appropriate regarding both the outcome and intervention (or exposure)?

3.3 Are there complete outcome data?

3.4 Are the confounders accounted for in the design and analysis?

3.5 During the study period, is the intervention administered (or exposure occurred) as intended?

4. Quantitative descriptive

4.1 Is the sampling strategy relevant to address the research question?

4.2 Is the sample representative of the target population?

4.3 Are the measurements appropriate?

4.4 Is the risk of nonresponse bias low?

4.5 Is the statistical analysis appropriate to answer the research question?

5. Mixed methods

5.1 Is there an adequate rationale for using a mixed methods design to address the research question?

5.2 Are the different components of the study effectively integrated to answer the research question?

5.3 Are the outputs of the integration of qualitative and quantitative components adequately interpreted?

5.4 Are divergences and inconsistencies between quantitative and qualitative results adequately addressed?

5.5 Do the different components of the study adhere to the quality criteria of each tradition of the methods involved?
